# Targeting the Gut Microbiome in Cardiovascular Disease: Current Therapeutic Strategies and Future Perspectives

**DOI:** 10.3390/nu18183091

**Published:** 2026-09-21

**Authors:** Diana Elena David, Cringuta Mariana Paraschiv, Laura Elisabeta Checherita, Stefan Andrei Chiriac, Gabriela Raluca Grigorasi, Vasile Valeriu Lupu, Ancuta Lupu, Leonard Iosif Pertea, Gabriela Paduraru, Anca Adam Raileanu, Elena Tataranu, Oana-Raluca Temneanu, Tatiana Dramba, Ana-Maria Casapu, Irina Mihaela Esanu

**Affiliations:** 1 Grigore T. Popa University of Medicine and Pharmacy, 700115 Iasi, Romania; diana-elena.david@d.umfiasi.ro (D.E.D.); laura.checherita@umfiasi.ro (L.E.C.); andrei-chiriac@umfiasi.ro (S.A.C.); gabriela-raluca.grigorasi@umfiasi.ro (G.R.G.); vasile.lupu@umfiasi.ro (V.V.L.); ancuta.ignat1@umfiasi.ro (A.L.); leonard.pertea@umfiasi.ro (L.I.P.); paduraru.gabriela@umfiasi.ro (G.P.); anca.adam-raileanu@umfiasi.ro (A.A.R.); temneanu.oana@umfiasi.ro (O.-R.T.); tatiana.dramba@email.umfiasi.ro (T.D.); anamaria.casapu@gmx.com (A.-M.C.); irina.esanu@umfiasi.ro (I.M.E.); 2 Faculty of Medicine and Biological Sciences, Stefan cel Mare University of Suceava, 720229 Suceava, Romania; elena.tataranu@usm.ro

**Keywords:** gut microbiome, cardiovascular disease, gut–heart axis, dysbiosis, microbial metabolites, trimethylamine-*N*-oxide, probiotics, prebiotics, pharmacomicrobiomics

## Abstract

The gut microbiota is increasingly recognized as an important modulator of cardiovascular health, demonstrating complicated interactions among diet, metabolism, immunology and host physiology. Evidence is accumulating that changes in the composition and function of the gut microbiome may contribute to the pathogenesis of atherosclerosis, hypertension, heart failure, thrombosis and other cardiovascular phenotypes through mechanisms involving microbial metabolites, intestinal barrier dysfunction, inflammation and metabolic dysregulation. In this context, the key question of this narrative review is: how far can modulation of the gut microbiome and its metabolic functions be a clinically meaningful approach in the prevention and treatment of cardiovascular disease, and which of the currently available microbiome-targeted approaches have sufficient mechanistic and clinical evidence to justify translation into cardiovascular practice? To answer this question, we review the evidence linking the gut microbiome to major cardiovascular phenotypes, such as atherosclerosis, hypertension, heart failure, thrombosis and atrial fibrillation, with particular focus on the mechanisms through which microbial metabolites, intestinal barrier dysfunction, inflammation and metabolic dysregulation may affect cardiovascular risk. We then review current and emerging strategies for microbiome modulation, including dietary interventions, prebiotics, probiotics, synbiotics, postbiotics, fecal microbiota transplantation and targeted inhibition of microbial metabolic pathways such as trimethylamine (TMA)/trimethylamine *N*-oxide (TMAO) production. We also examine bidirectional interactions between cardiovascular drugs and gut microbiome and the role of these interactions in the development of precision methods to cardiovascular prevention and treatment. Finally, we outline the major limitations of the current evidence and highlight goals for future mechanistic, translational and clinical research.

## 1. Introduction

### 1.1. Cardiovascular Disease: A Growing Global Health Challenge

Cardiovascular diseases (CVDs) are the leading global causes of morbidity and mortality, accounting for nearly one-third of all deaths and placing a significant socioeconomic burden on healthcare systems [1]. Advances in prevention, pharmacological treatment and interventional cardiology have resulted in decreased age-standardized cardiovascular mortality in many high-income countries. However, the absolute burden of CVD is still increasing, mainly due to population aging and the rising prevalence of obesity, type 2 diabetes mellitus, hypertension and metabolic syndrome [2]. Furthermore, a large proportion of patients still experience cardiovascular events despite excellent therapy of recognized risk factors, underlining the importance of residual cardiovascular risk, as well as the need to find other modifiable determinants of disease [2].

Within this framework, the gut microbiome has been recognized as a key regulator of human health and disease and has extended the definition of cardiovascular risk beyond host genetics and conventional environmental effects [3,4]. The human gastrointestinal (GI) tract contains a complex community of microbes, primarily bacteria but also archaea, viruses and fungi, whose collective metabolic potential plays a role in nutrient metabolism, vitamin production, transformation of bile acids, the integrity of the intestinal barrier, and regulation of innate and adaptive immune responses [5,6,7]. Changes in the composition and functional capabilities of this microbial population, usually referred to as gut dysbiosis, have been linked to metabolic disorders and cardiovascular diseases [7,8,9]. Notably, the concept of functional dysbiosis, referring to changes in microbial metabolic activity or function, may be particularly significant in the setting of cardiovascular disease, as microbial metabolites can exert direct effects on host metabolic, inflammatory, vascular and immunological pathways.

Evidence from experimental and clinical studies revealing bidirectional interactions between the intestinal microbiota and the cardiovascular system through metabolic, inflammatory, endocrine, and immune-mediated pathways strengthened the concept of the gut–heart axis [3,10]. Recent work has advanced this notion from descriptive connections of microbial composition with cardiovascular disease to the discovery of microbial pathways and functions that may impact cardiovascular phenotypes. In a multi-omic analysis of 1429 individuals from the Framingham Heart Study, integrated metagenomic and metabolomic profiling identified microbial pathways associated with cardiovascular health, such as cholesterol metabolism, with species belonging to the genus *Oscillibacter* being associated with lower fecal and plasma cholesterol concentrations and conserved cholesterol-metabolizing capabilities in vitro [11]. These results highlight the growing importance of microbial activity and host–microbe metabolic interactions in addition to taxonomic composition.

### 1.2. Dysbiosis as an Emerging Cardiovascular Risk Factor

Several mechanisms can explain the link between gut dysbiosis and cardiovascular disease, such as variations in the generation of microbial metabolites, intestinal barrier dysfunction, immunological activation, and systemic inflammation [8,9,12]. Trimethylamine *N*-oxide (TMAO) is one of the most studied cardiovascular risk factors among microbial metabolites. TMAO is produced when gut microbes convert dietary choline, phosphatidylcholine, and L-carnitine into trimethylamine, which is then oxidized by the liver. Elevated TMAO levels have been linked to accelerated atherosclerosis, endothelial dysfunction, increased platelet reactivity, unfavorable cardiac remodeling and higher risk for significant adverse cardiovascular events [12,13,14]. These correlations are actively pursued to determine their strength and causality, but experimental and clinical data suggests an essential role of the gut microbial TMA–TMAO pathway in cardiovascular pathology [12,13,14].

In contrast, short-chain fatty acids (SCFAs), such as acetate, propionate and butyrate, are mainly produced by bacterial fermentation of dietary fibers and may have beneficial (but context-dependent) cardiometabolic effects [15]. SCFAs have a role in the regulation of blood pressure, glucose and lipid metabolism, immune-cell differentiation and inflammatory signaling and preserve the integrity of the intestinal barrier [15]. Another important mechanism for the impact of the gut microbiota on cardiovascular disease is through changes in bile acid metabolism. Gut microorganisms convert primary bile acids to secondary bile acids that signal to the host via receptors, such as farnesoid X receptor (FXR) and G protein-coupled bile acid receptor 1 (TGR5), and influence lipid and glucose metabolism, energy homeostasis, and inflammatory pathways [16,17]. These microbial metabolites are key mediators of host–microbiome interactions and prospective therapeutic targets.

Apart from the metabolic abnormalities, gut dysbiosis could also disrupt the integrity of the intestinal epithelial barrier allowing the transfer of bacterial components like lipopolysaccharide (LPS) to the systemic circulation. This mechanism, commonly referred to as metabolic endotoxemia, can activate pattern-recognition receptors and induce persistent low-grade inflammation that can contribute to endothelial dysfunction, oxidative stress, vascular remodeling, and progression of atherosclerotic lesions [18,19]. The gut microbiome has also been increasingly implicated in hypertension, heart failure, and thromboembolic illness, via complicated interactions, including immunological control, autonomic pathways, intestinal permeability, and altered synthesis of microbial metabolites [20,21,22]. In particular, recent studies suggest that microbial metabolites and intestinal barrier dysfunction affect platelet reactivity and vascular inflammatory pathways, validating a broader role of the gut microbiome in thrombotic cardiovascular phenotypes [22].

Combined, these studies have transformed the gut microbiota from a possible marker of cardiovascular disease to a potentially changeable determinant and therapeutic target. There has, consequently, been increased focus on ways to modulate microbial composition or function, including dietary manipulation, probiotics, prebiotics, synbiotics, postbiotics, and pharmaceutical modulation of host–microbiome interactions. However, there are still substantial translational barriers, including the distinction between association and causation, high interindividual variability in microbial composition and metabolic responses and the need for more solid evidence for the clinical efficacy of microbiome-targeted treatments. These factors give a justification to evaluate current information on microbiome-derived processes in cardiovascular disease, treatment methods available and their possible translational implications.

**Methods.** This article provides a narrative review of current research on the involvement of the gut microbiome in cardiovascular disease, concentrating on microbiome-derived mechanisms, therapeutic approaches and translational implications. We searched the literature in PubMed and Web of Science from inception to August 2026. The search strategy was meant to capture literature on gut microbiota and cardiovascular disease connection, microbiome-derived metabolites and processes, dietary and pharmacological interactions and microbiome-targeted therapeutic approaches. The search strategy included combinations of the following keywords and associated terms: “gut microbiota”, “gut microbiome”, “dysbiosis”, “cardiovascular disease”, “atherosclerosis”, “hypertension”, “heart failure”, “atrial fibrillation”, “microbial metabolites”, “trimethylamine”, “trimethylamine *N*-oxide”, “TMAO”, “short-chain fatty acids”, “bile acids”, “nutrition”, “dietary fiber”, “Mediterranean diet”, “polyphenols”, “probiotics”, “prebiotics”, “synbiotics”, “postbiotics”, “fecal microbiota transplantation” and “microbiome-targeted treatments”. These phrases were combined using Boolean operators (AND and OR) in the structure (“gut microbiome” OR “gut microbiota” OR dysbiosis) AND (“cardiovascular disease” OR atherosclerosis OR hypertension OR “heart failure” OR “atrial fibrillation”), with other combinations tailored to the specific topics discussed in the review.

Publications were considered eligible if they examined the association between the gut microbiota/microbiome and cardiovascular disease or if they provided mechanistic, clinical, or translational evidence relevant to microbiota-derived metabolites, cardiovascular drugs, dietary interventions, or microbiome-targeted treatments. Microbiota-derived metabolites and pathways implicated in cardiovascular disease, interactions between the gut microbiome and cardiovascular drugs, dietary interventions that impact the gut microbiome and cardiovascular outcomes, and therapeutic approaches targeting the microbiome were included in the eligible literature.

Publications were eliminated if they did not directly address the gut microbiome–cardiovascular disease connection, cardiovascular outcomes, microbiota-derived processes or metabolites, or the specific objectives of the present review. Studies were also excluded if the complete text was unavailable or the publication did not contain adequate information to assess its relevance to the aims of the review.

Although no formal systematic review quality-appraisal tool was applied, given the narrative nature of this review, methodological quality was considered qualitatively when interpreting and weighting evidence. The literature reviewed included a variety of study designs, including randomized controlled trials, systematic reviews and meta-analyses, prospective cohort studies, mechanistic and animal studies, and expert consensus statements. When available, randomized controlled trials, large prospective cohort studies, and systematic reviews or meta-analyses were prioritized over smaller observational or cross-sectional studies in considering the strength of evidence for a particular mechanism or intervention. Consensus and position statements from recognized scientific bodies were used to anchor terminology and definitions rather than as primary evidence of clinical efficacy.

## 2. The Gut–Heart Axis: Mechanisms Linking Gut Dysbiosis to Cardiovascular Disease

### 2.1. Gut Microbiota in Cardiovascular Homeostasis

The human gastrointestinal (GI) tract contains a complex and metabolically active microbial community that cohabits in a dynamic symbiotic relationship with the host. The structure of the gut microbiota varies greatly among individuals and is influenced by factors such as age, genetics, food, lifestyle, medication usage, and environmental exposures, but its functional ability is surprisingly similar. Such functional redundancy allows the gut microbiota to retain key physiological functions despite interindividual taxonomic diversity. Intestinal bacteria are not simply commensal organisms but rather an integrated metabolic and immunological organ that produces a vast diversity of bioactive compounds that affect local intestine physiology and distant organs, including the cardiovascular system [23,24].

The gut microbiome modulates cardiovascular homeostasis via several complementary mechanisms. It participates in the fermentation of non-digestible dietary carbohydrates, resulting in the production of short-chain fatty acids (SCFAs), serving as energy sources for colonocytes and modulating the integrity of the intestinal barrier, immune tolerance and host metabolism [25]. At the same time, gut microbes metabolize bile acids, amino acids and other food components, consequently affecting cholesterol metabolism, glucose balance, vascular function and systemic inflammatory responses [26]. In addition, the gut microbiota contributes to the maturation of both innate and adaptive immunity by maintaining the equilibrium of pro- and anti-inflammatory immune cell populations. It also preserves the intestinal epithelial barrier integrity by regulating the expression of tight junction proteins and mucus production. These physiological processes collectively provide the gut microbiota with a fundamental role in the regulation of metabolic and immunological homeostasis, therefore providing the biological basis for the developing role of the microbiota in cardiovascular health and illness [27,28].

Gut dysbiosis is defined as a disruption of this delicate balance and is characterized by changes in the composition of the microbiota and functional changes in the microbial metabolic activities. Increasing data suggests that dysbiosis is characterized by low microbial diversity, loss of helpful commensal bacteria, decreased production of protective metabolites and proliferation of potentially dangerous microbes. Such modifications affect host–microbiome interactions and activate metabolic and inflammatory pathways implicated in cardiovascular disease [29,30]. Understanding the physiological significance of the healthy gut microbiota is thus fundamental for exploring the potential of targeted regulation of the microbial populations as a novel therapeutic approach for cardiovascular prevention and treatment.

Among the many mechanisms that relate gut dysbiosis to cardiovascular disease, changes in microbial metabolite production, disturbance of intestinal barrier integrity and dysregulated immunological signaling have emerged as the main pathways of the gut–heart axis.

### 2.2. Gut Dysbiosis: Definition, Determinants, and Functional Consequences

The gut microbiota is a dynamic ecology constantly responding to environmental and host-derived variables. Under physiological circumstances, microbial populations establish a symbiotic relationship with the host, which is involved in the maintenance of metabolic homeostasis, regulation of immunity and the integrity of the intestinal barrier [31]. However, a perturbation of this balance can lead to gut dysbiosis, a condition defined by alterations of the composition, variety and functional capability of the intestinal microbiota [32]. In addition, dysbiosis should not be considered only as a taxonomic imbalance but as a perturbation of microbial functions that impairs host–microbiome interactions and drives disease pathogenesis. Contemporary microbiome research is increasingly emphasizing the concept of functional dysbiosis, in which changes in microbial metabolic activity may be the cause or even more important than observable changes in microbial composition [33].

Gut dysbiosis is triggered by the complex interaction of several host and environmental factors. Diet is one of the most powerful determinants of microbial ecology, and western dietary patterns high in saturated fat, refined carbohydrates, and ultra-processed foods are consistently associated with reduced microbial diversity and reduced production of beneficial microbial metabolites [34]. In contrast, diets high in dietary fiber, polyphenols from plants and fermented foods favor the growth of saccharolytic bacteria that can produce short-chain fatty acids and other metabolites associated with cardiovascular protection [35,36]. Dysbiosis has been implicated not just in the pathogenesis of diet but also aging, obesity, type 2 diabetes mellitus, chronic renal disease, smoking, alcohol intake, physical inactivity and psychological stress. Pharmacological medications, including antibiotics, proton pump inhibitors, metformin and cardiovascular medicines that are increasingly identified, can further modulate microbial communities by modifying bacterial composition and metabolic activity [37]. These results underline the very dynamic character of the gut microbiome and reinforce the idea that microbial composition might be therapeutically modulated throughout life.

Generally, dysbiosis is not characterized by a single disease-specific microbial signature but by reduced microbial diversity, loss of health-associated commensal microorganisms, expansion of opportunistic pathobionts and impaired production of metabolites involved in maintaining intestinal and systemic homeostasis [31]. These changes impair epithelial barrier function, alter metabolism of bile acids and amino acids, reduce synthesis of short-chain fatty acids and induce persistent low-grade inflammation via activation of innate and adaptive immunological mechanisms. The gut dysbiosis has emerged as a key component of the gut–heart axis, as these functional deficiencies have direct effects on vascular biology, lipid metabolism, glucose homeostasis, and immunological responses [38,39]. Subsequent sections highlight the key molecular routes by which microbial dysregulation contributes to cardiovascular disease and the biological basis for microbiome-targeted therapeutics.

### 2.3. Major Mechanisms Linking Gut Dysbiosis to Cardiovascular Disease

Gut dysbiosis may play a role in cardiovascular disease (CVD) through several interrelated metabolic, inflammatory and vascular pathways. Changes in microbial composition and function can impact the generation of bioactive metabolites, affect host metabolic signaling, disrupt intestinal barrier integrity and increase systemic immunological activation. The best characterized mechanisms comprise microbial transformation of dietary nutrients to trimethylamine *N*-oxide (TMAO), altered production of short-chain fatty acids (SCFAs), disruption of bile acid metabolism, intestinal barrier dysfunction with subsequent exposure to microbial products and changes in amino acid-derived metabolites. These processes should not be considered isolated, but as part of a complex network in which nutrition, microbial metabolism, host physiology and cardiovascular risk interact.

#### 2.3.1. Trimethylamine *N*-Oxide (TMAO)

One of the most well-studied pathways linking gut microbiota to CVD is the trimethylamine (TMA)–TMAO pathway. Intestinal bacteria convert food substrates containing trimethylamine groups (phosphatidylcholine, choline and L-carnitine) to TMA, which enters the portal circulation and is oxidized mainly in the liver by flavin-containing monooxygenase 3 (FMO3) to produce TMAO. Thus, circulating TMAO concentration is not a direct readout of microbial activity per se, but rather reflects the combined influence of dietary substrate availability, microbial metabolic capacity, intestinal absorption, and host FMO3 activity, the latter of which varies substantially between individuals due to genetic polymorphisms, sex (estrogen suppresses FMO3 expression), and diet. This host-level variability is an underappreciated confounder in TMAO research: two individuals with identical microbial TMA-producing capacity could have dramatically different circulating TMAO, meaning that TMAO-outcome associations conflate microbial and host metabolic contributions in ways that current studies rarely distinguish apart.

Experimental and clinical observational studies have hypothesized that TMAO might modulate pathways relevant to atherosclerosis and thrombosis. In animal models, elevated TMAO exposure has been linked to changes in cholesterol metabolism, decreased reverse cholesterol transfer, vascular inflammation and faster development of atherosclerotic lesions. Furthermore, experimental studies demonstrated that TMAO might induce platelet hyperreactivity and increase thrombosis potential [40]. Associations between circulating TMAO concentrations and cardiovascular or thrombotic events have been observed in humans in observational studies, including reporting from prospective cohorts and meta-analyses [41]. However, these human data do not prove that TMAO is a causal factor of cardiovascular disease. The discovered relationships could be impacted by renal function, dietary patterns, metabolic status and other clinical variables. Importantly, there are currently no clinical trials showing that pharmacological or microbiome-directed decrease of TMAO improves cardiovascular outcomes. Thereby, TMAO should be considered as a biologically plausible mediator and possible risk marker, although its causative and therapeutic relevance in humans is not fully demonstrated.

Nevertheless, the TMAO pathway provides an important proof of concept for the gut–heart axis: a dietary nutrient can be transformed by gut microorganisms into a circulating molecule that may affect vascular and thrombotic function. As a consequence, this route has drawn substantial interest as a therapeutic target, including strategies to manipulate food substrate availability, inhibit microbial TMA biosynthesis, modulate microbial enzymes implicated in TMA biosynthesis, or modulate host TMAO production. However, these treatments are still exploratory and have not yet been shown to minimize cardiovascular events in humans in clinical outcome trials.

#### 2.3.2. Short-Chain Fatty Acids

Dietary carbohydrates escaping digestion in the upper gastrointestinal system are fermented by gut microorganisms to short-chain fatty acids (SCFAs), mainly acetate, propionate and butyrate. SCFAs represent a critical link between microbial metabolism and host physiology, with reported involvement in intestinal epithelial integrity, immunological modulation, glucose and lipid metabolism, and vascular function. Mechanistically, SCFAs exert their functions through at least two separate mechanisms: receptor-mediated signaling by the free fatty acid receptors FFAR2 (GPR43) and FFAR3 (GPR41) and receptor-independent epigenetic regulation through inhibition of histone deacetylases (HDACs) [42]. These two mechanisms are not interchangeable—FFAR2/FFAR3 signaling operates at physiological SCFA concentrations and mediates rapid, receptor-specific effects on immune cell function and metabolic hormone secretion, whereas HDAC inhibition requires the higher intracellular SCFA concentrations (particularly of butyrate) typical of the colonic lumen, and produces slower, transcription-wide effects on gene expression [43]. Thus, identifying the dominant mechanism in a given tissue or physiological setting is not a trivial technical point, but rather affects whether an observed SCFA effect is to be regarded as a targeted signaling event or a more global epigenetic consequence of local SCFA build-up.

However, butyrate is distinctive in this context, as it is the major energy source for colonocytes through β-oxidation, in addition to its receptor and epigenetic function [43]. This dual identity as a fuel and a signaling/epigenetic molecule has an important experimental implication that is often underappreciated: the HDAC-inhibitory effect of butyrate is most apparent when its role as an energy substrate is diminished, as in colonocytes undergoing a Warburg-like shift to glucose metabolism, wherein unmetabolized butyrate accumulates intracellularly and more readily inhibits HDAC activity. Consequently, the major physiological effect of butyrate (providing energy) and its major experimental/pathological effect (HDAC inhibition) may be different metabolic states of the same cell rather than two independent, additive actions—a distinction that the current microbiome–cardiovascular literature rarely makes when attributing cardiovascular effects to “butyrate signaling.”

Additional experimental work, especially in animal models, suggests that signaling by SCFAs may affect vascular tone and blood pressure [44]. However, the cardiovascular relevance of SCFAs is less well established in people than the experimental literature may imply. Their effects are likely to be dependent on the particular SCFA, concentration, place of synthesis, receptor expression, tissue distribution, and metabolic environment. Furthermore, fecal and circulatory levels of SCFAs are simply surrogate markers of microbial production and biological activity. Associations of SCFA concentrations with cardiovascular phenotypes cannot in themselves prove that changed SCFA production is the cause of cardiovascular illness.

This distinction is relevant when evaluating dietary fiber as a potential microbiome-mediated therapeutic. While some of the cardiovascular benefits ascribed to higher fiber intake may be plausibly mediated by microbial fermentation and SCFA signaling, dietary fiber also impacts many microbial and host pathways simultaneously. These effects may be further modified by interindividual changes in baseline microbiota, substrate availability, microbial metabolic capability, and host responsiveness. Consequently, total SCFA concentrations should not be considered as a validated microbiome-targeted approach for cardiovascular protection. Future therapies may need to target specific microbial roles, metabolic pathways or host–microbiome interactions rather than generic increases in overall SCFA production.

#### 2.3.3. Altered Bile Acid Metabolism and Signaling

##### Microbial Transformation and Bile Acid Signaling

Another important metabolic link between gut microbiota and cardiovascular physiology is bile acids. Primary bile acids are generated from cholesterol in the liver and released into the intestine, where they are modified by intestinal bacteria. These microorganisms deconjugate primary bile acids and also carry out other reactions that produce secondary and other structurally different bile acids. These microbial modifications greatly increase the bile acid pool and alter its physiologic signaling properties.

In addition to their long-standing role in lipid digestion, bile acids also function as signaling agents through receptors, including the farnesoid X receptor (FXR) and the Takeda G-protein-coupled bile acid receptor 1 (TGR5). Activation of these receptors influences lipid and glucose metabolism, energy consumption, inflammatory responses and other activities crucial to cardiometabolic health. However, not all effects are positive and the direction of the effect appears to be tissue-specific and context-dependent. Intestinal FXR activation is generally considered metabolically protective, yet systemic pharmacologic FXR agonism in humans has produced the opposite lipid signature: in the FLINT trial, the FXR agonist obeticholic acid increased LDL cholesterol and decreased HDL cholesterol compared with placebo, an effect attributed to decreased hepatic LDL receptor expression [45]. This immediately shows that FXR activation cannot be assumed a priori to be cardioprotective because the same receptor pathway might plausibly change cardiovascular risk in either direction depending on where and how it is activated. Therefore, dysbiosis-related changes in microbial bile acid transformation may alter host metabolic signaling despite relatively stable total bile acid concentrations.

##### Evidence Linking Bile Acids to Cardiovascular Disease

Emerging evidence in humans suggests that variations in bile acid content and signaling may be connected with cardiometabolic risk. For example, a prospective cohort study of human patients with newly diagnosed type 2 diabetes found that circulating secondary bile acids and genetic variation in bile acid receptors were associated with subsequent cardiovascular risk, suggesting that both metabolite composition and host receptor biology may contribute to cardiovascular phenotypes [46]. This association, however, is based on an observational design in a population with perturbed bile acid metabolism by diabetes and glycemic status, and so reverse causality and residual confounding by metabolic disease severity cannot be excluded; the study shows correlation of bile acid biology with risk, not that bile acid changes are causally upstream of it. Furthermore, randomized dietary intervention results in humans suggest that gut microbial bile acid metabolism may modulate the cardiometabolic response to a Mediterranean diet intervention [47]. However, these results do not indicate that microbiota-dependent changes in bile acid metabolism are a direct cause of cardiovascular illness, nor do they illustrate that therapeutic manipulation of specific bile acid pathways improves cardiovascular outcomes. Further mechanistic support is provided by experimental studies of FXR- and TGR5-dependent signaling, but the extent to which these processes transfer to clinically relevant cardiovascular consequences in humans is unclear.

Crucially, bile acids should not be perceived as either “protective” or “harmful.” The effects of these substances rely on their chemical structure, concentration, tissue distribution and receptor-specific signaling. Thus, cardiovascular effects of dysbiosis may result from changes in the composition and signaling qualities of the bile acid pool, rather than a simple increase or reduction in the total amount of bile acids produced. This complexity is particularly relevant when considering treatment techniques aiming at changing microbial bile acid metabolism.

The mechanisms connecting gut microbial dysbiosis to cardiovascular disease should be interpreted overall, depending on the level of evidence supporting each pathway. Mechanistic and observational data are currently relatively strong supporting the TMA/TMAO axis, but definitive proof that lowering TMAO improves cardiovascular outcomes in humans is still missing. Biological and experimental data strongly support SCFA signaling and microbiota-dependent bile acid metabolism, although their cardiovascular effects in humans are apparently more varied and context-dependent. The presence of a biologically plausible microbiome–cardiovascular link should not be taken as endorsement of that pathway as a clinical treatment target. Such distinction is particularly significant when evaluating microbiome-directed therapies, when improvement in microbial composition or metabolite profiles does not always translate into improved cardiovascular outcomes.

#### 2.3.4. Intestinal Barrier Dysfunction and Metabolic Endotoxemia

The intestinal barrier is a fundamental interface between the microbial environment and the systemic circulation. It consists of the mucus layer, epithelial cells, tight-junction complexes, immunological components and other defense systems that jointly govern the passage of nutrients while limiting the translocation of microbes and possibly dangerous microbial products. These processes can be impaired by dysbiosis, nutrition, metabolic abnormalities and intestinal inflammation, leading to increased intestinal permeability.

One such hypothesized result is increased systemic exposure to lipopolysaccharide (LPS), a structural component of the Gram-negative bacterial outer membrane. The term “metabolic endotoxemia” refers to a chronic, low-grade increase in circulating endotoxin that is related to metabolic illness. In a physiologically viable process supported by experimental studies, LPS promotes innate immune signaling via toll-like receptor 4 (TLR4), with downstream NF-κB activation implicated in endothelial activation, oxidative stress, insulin resistance and vascular inflammation [48]. An important limitation, however, is that much of this mechanistic evidence is derived from experimental LPS exposures, whether in cell culture or animal models, that do not necessarily replicate the low-grade, chronic concentration range implicated by “metabolic endotoxemia” in humans. Some of the foundational studies, instead, employed acute, high-dose LPS challenge paradigms that are closer to models of sepsis than of chronic low-grade endotoxemia. The relevance of such mechanistic discoveries from these experiments to the much lower, chronic exposures relevant to cardiometabolic disease has not been thoroughly proven, and this distinction should moderate the directness of extrapolation of these experimental data to the human state.

The role of gut barrier disruption may not be restricted to LPS alone. Increased permeability may result in exposure to a wider range of microbial-associated molecular patterns and metabolites and increase systemic immunological activation. In addition, there could be a bidirectional relationship where dysbiosis leads to a decrease in the production of microbial metabolites that maintain the barrier, reduced barrier function increases host exposure to microbial products, and consequent inflammation further compromises intestinal integrity. Thus, intestinal permeability may be an essential mediator between microbial dysbiosis and systemic cardiovascular inflammation.

In human studies, the idea of metabolic endotoxemia should be interpreted with caution. Circulating LPS is technically difficult to assess, and existing techniques may be subject to sample handling, dietary lipids and other methodological problems. Thus, although there is experimental evidence consistent with a biologically plausible function of microbial products in systemic inflammation, the contribution of circulating LPS itself to the pathogenesis of human CVD remains uncertain. This distinction is crucial because the connection between an LPS-related parameter and cardiovascular risk should not necessarily be considered evidence of a direct causative pathway [49].

Apart from TMAO, SCFAs and bile acids, various other microbiota-derived metabolites, including indole derivatives, phenylacetylglutamine (PAGln), hydrogen sulfide, and branched-chain amino acids, have also been connected with cardiovascular abnormalities as detailed in the following sections. For each, the data is far more varied than for the TMAO or SCFA pathways, and in many situations, it remains impossible to separate a direct microbial metabolic contribution from broader modifications in host metabolism that co-occur with dysbiosis rather than being triggered by it.

#### 2.3.5. Other Microbial Metabolites

Indole metabolites. Other microbiota-derived metabolites, in addition to TMAO, SCFAs and bile acids, may alter cardiovascular function. These substances are largely generated by microbial metabolism of dietary amino acids and other host- or diet-derived substrates. The evidence foundation for some metabolites is less developed, although several routes have been explored for their possible impact on atherosclerosis, endothelial function, inflammation and thrombosis. Microbes in the gut convert the dietary compound tryptophan to numerous indole derivatives, including indole-3-propionic acid (IPA), indole-3-acetic acid and other indoles. These metabolites can impact intestinal barrier function, oxidative stress and immunological responses and can modulate host signaling pathways, such as the aryl hydrocarbon receptor. Cardiovascular effects of indole metabolites appear to be metabolite rather than class specific. The human observational evidence from a combined microbiome–metabolome investigation shows that circulating IPA concentrations were lower in patients with coronary artery disease, and lower levels of IPA were linked with increased atherosclerotic cardiovascular disease burden. By contrast, experimental investigations in mice have shown that supplementation with IPA reduces the production of atherosclerotic lesions. Mechanistic studies have suggested that IPA may enhance macrophage cholesterol efflux and reverse cholesterol transport through modulation of the miR-142-5p/ABCA1 pathway [50]. The findings are consistent with the biological plausibility of a protective role of IPA and other potentially beneficial microbial metabolites, but do not demonstrate reduced IPA availability as a causal driver of cardiovascular disease in people.

Phenylacetylglutamine (PAGln). For example, PAGln is a microbiota-dependent molecule that could relate intestinal amino acid metabolism to thrombosis. Phenylalanine is converted by gut microorganisms to phenylacetic acid, which is then conjugated by the host to PAGln. Higher circulating PAGln concentrations have been associated with cardiovascular disease and worse cardiovascular outcomes in human observational studies. Mechanistic evidence has also been shown by experimental research for an increase in platelet activation and thrombotic potential by PAGln. Mechanistically, PAGln can activate adrenergic G-protein-coupled receptors, including α2A-, α2B- and β2-adrenergic receptors, offering a potential method by which microbial metabolism may alter platelet function [51]. These findings provide support for a physiologically plausible gut–thrombosis axis and imply that microbial processes contributing to PAGln production may be possible therapeutic targets. However, it has not been demonstrated in clinical trials that lowering PAGln synthesis or signaling can reduce thrombotic or cardiovascular events in humans.

Hydrogen sulfide. Hydrogen sulfide (H_2_S) is a gaseous signaling molecule generated endogenously by mammalian tissues and intestinal bacteria. Its cardiovascular activities include regulation of vascular tone, oxidative stress, inflammation, vascular smooth-muscle behavior and platelet activity. The microbial generation of H_2_S depends on the availability of sulfur-containing substrates and the abundance and metabolic activity of sulfur-metabolizing bacteria. Preclinical and experimental studies suggest that physiological concentrations of H_2_S may exert vascular-protective effects, whereas excessive exposure may produce adverse cellular effects. Thus, like with certain other microbial metabolites, its biological action seemed to be concentration- and context-dependent. However, there is a lack of direct data linking changes in intestinal H_2_S production with clinical cardiovascular outcomes, but the available evidence supports a potential role for microbiota-derived H_2_S in vascular homeostasis and atherosclerosis [52].

Branched-chain amino acids. Leucine, isoleucine and valine are essential amino acids and are involved in protein synthesis, energy metabolism and nutrient-sensing pathways. Elevated circulating levels of BCAAs have been related to insulin resistance, obesity and other metabolic disorders, and recent data has demonstrated a link between BCAA dysregulation and CVD. Human observational data is from a large prospective cohort study of 27,041 women in which elevated circulating BCAA concentrations were related to the incidence of CVD despite adjustment for recognized cardiovascular risk factors [53]. Similar relationships have been identified in other populations, such as patients with chronic renal disease and hypertension. However, circulating BCAA concentrations may be affected by food intake, host catabolism, insulin sensitivity, mitochondrial metabolism and potentially microbial metabolism, and it is therefore difficult to directly relate their cardiovascular connections to gut microbial activity. Accordingly, BCAAs should not currently be interpreted as specific biomarkers of gut microbial dysfunction. In addition, Mendelian randomization studies have not consistently demonstrated a causal connection between genetically predicted circulating BCAA concentrations and cardiovascular characteristics [54]. Therefore, BCAAs may represent metabolic indicators and potential mediators of cardiometabolic dysfunction, but the causal role of BCAAs in CVD and, specifically, the extent to which gut microbial metabolism contributes to these correlations, needs further clarification. (Summary in Table 1).

In general, the data available indicates that the cardiovascular effects linked to gut dysbiosis are unlikely to be caused by a single microbial taxon or molecule. Rather, dysbiosis could be defined by concurrent shifts in microbial metabolic pathways, including increased production of potentially pathogenic metabolites, decreased bioavailability or altered signaling of other microbial products, disturbance of bile acid metabolism, perturbation of intestinal barrier function, and modulation of systemic inflammatory and thrombotic pathways. This supports a functional rather than a compositional view of the gut microbiome–cardiovascular link.

But importantly, the strength of evidence for these paths varies greatly. One of the more extensively studied pathways both experimentally and epidemiologically is the TMA/TMAO axis, although whether TMAO is a causal mediator and if reducing TMAO will improve cardiovascular outcomes in humans remain open questions. Evidence for SCFA signaling, bile acid metabolism, indole derivatives, H_2_S, PAGln and BCAAs is similarly variable and differs greatly between experimental and human research. Specifically, relationships between circulating metabolites and cardiovascular phenotypes do not indicate that altered microbial production is accountable for the observed clinical consequences.

Accordingly, the existence of a biologically plausible gut microbiome–cardiovascular connection should not be considered as evidence that the pathway is an established therapeutic target. Future investigations should include longitudinal and mechanistic studies in humans, consistent methods for microbiome and metabolite evaluation, and appropriately powered microbiome-targeted clinical trials with clinically meaningful cardiovascular outcomes. Such research will be essential to understand which microbiome-associated changes are causal processes, disease-associated biomarkers or modifiable treatment targets.

### 2.4. Gut Dysbiosis Across the Cardiovascular Disease Spectrum

The link between gut dysbiosis and cardiovascular disease is not limited to a cardiovascular phenotype. Alterations of gut microbial composition and function have been described in atherosclerosis, hypertension, heart failure (HF), atrial fibrillation (AF) and ischemic stroke. While the exact microbial signatures differ between diseases and study populations, some common patterns are apparent, including a decreased abundance of short-chain fatty acid (SCFA)-producing microorganisms, an increase in potentially pathogenic or pro-inflammatory taxa, alterations in microbial metabolism of amino acids and bile acids, a compromised intestinal barrier function, and increased systemic inflammatory signaling. Most human studies now available are observational, and microbiome profiles are altered by age, food, usage of medications, renal function, metabolic condition and disease severity. Thus, microbial fingerprints associated with disease should not be automatically considered causal drivers.

#### 2.4.1. Atherosclerosis

Among cardiovascular disorders, the gut microbiota has been most studied in atherosclerosis. Initial metagenomic investigations revealed changes in the composition of gut microbial communities between symptomatic atherosclerosis patients and healthy controls. For instance, *Collinsella* was concentrated in individuals with symptomatic atherosclerosis while *Roseburia* and *Eubacterium* were enriched in controls. Moreover, functional variations in the gut metagenome were also found, indicating that changes in microbial metabolic ability may have more biological implications than taxonomic differences per se [55].

Additional studies have connected atherosclerotic disease with numerous microbiome-related processes, including TMAO-related metabolism, SCFA synthesis, bile acid metabolism, and other microbial metabolites. These pathways may affect cholesterol management, macrophage function, endothelial activation, inflammatory signaling and platelet reactivity. Conversely, a reduction of SCFA-producing microbes may compromise gut barrier integrity and immunological function. Increased intestinal permeability and exposure to microbial compounds such as lipopolysaccharide have also been suggested to contribute to vascular inflammation.

However, the variability of microbial observations amongst human cohorts challenges the identification of a universal microbial signature related to atherosclerosis. Observed diversity may be explained by differences in diet, geography, drug exposure, illness phenotype, comorbidity load, sequencing technology and analytical methodologies [56]. There is, therefore, no evidence to date for the use of any single microbial taxon as a viable clinical biomarker for atherosclerosis. Instead, these findings increasingly lend themselves to the study of functional microbial pathways and metabolite synthesis. Whether such changes are causes, outcomes or moderators of atherosclerotic disease must be determined.

#### 2.4.2. Hypertension

Changes in gut microbial diversity and composition have also been linked to hypertension. In a previous metagenomic study of 60 patients with primary hypertension and 60 matched controls, decreased microbial diversity and altered microbial gene profiles were found in persons with hypertension. Opportunistic taxa such as *Klebsiella* and *Streptococcus* were more prevalent in the hypertensive group, while SCFA-producing bacteria such as *Roseburia* and *Faecalibacterium prausnitzii* were more abundant in controls [57]. Later research and systematic analyses have, on the whole, indicated a link between altered microbial diversity and hypertension, but the direction and size of alterations in individual taxa have differed greatly between studies [58]. Several plausible pathways might link these changes to blood pressure regulation, including SCFA-mediated signaling, modulation of vascular tone, immunological activation and changes in intestinal barrier function. Experimental studies provide mechanistic support for a function of microbial metabolites in blood pressure regulation, while evidence from human research remains less consistent. In particular, diminished microbial diversity or depletion of certain SCFA-producing taxa cannot be regarded at present as distinct biomarkers or known causes of hypertension. Dietary patterns, antihypertensive medicines, obesity, renal function and other metabolic variables may independently affect both the gut flora and blood pressure.

Accordingly, current evidence supports an association between gut microbial alterations and hypertension but does not establish a uniform microbial signature or a causal pathway that can be therapeutically targeted in clinical practice.

#### 2.4.3. Heart Failure

Heart failure involves complex bidirectional interactions between the circulatory system and the gut. Dysbiosis is common in HF patients, with some cohorts showing reduced microbial diversity, reduction of SCFA-producing bacteria and concentration of potentially pathogenic taxa. One metagenomic investigation showed that HF patients had increased abundance of microbial genes involved in TMA production, and the abundance of cntA/B genes was closely associated with circulating TMAO concentrations; these genes were mainly assigned to *Escherichia* and *Klebsiella* species [59]. This finding should be interpreted with an important limitation: a bloom of *Enterobacteriaceae*, including these genera, is a well-established, non-specific signature of gut dysbiosis across many states of critical or chronic illness, reduced gut perfusion, and antibiotic exposure—it is not unique to HF. As a single cross-sectional investigation, this work reveals a plausible mechanistic relationship between microbial functional capacity and circulating TMAO, but cannot establish whether the dysbiosis identified is HF-specific or a generic indicator of illness severity.

The reverse causal path is also possible: the pathophysiology of HF may independently contribute to intestinal dysbiosis. Changes in dietary intake, intestinal perfusion, venous congestion, intestinal edema, and decreased cardiac output may all impact microbial ecology and alter intestinal barrier function. Increased gut permeability may facilitate transfer of microbial metabolites and contribute to systemic inflammation, thereby perpetuating a self-sustaining loop of gut dysfunction and cardiac deterioration. Much of the molecular complexity of this reverse pathway comes from physiological thinking and animal or experimental congestion models rather than direct human confirmation and should be regarded as a probable complementary mechanism rather than an established one.

Higher circulating TMAO has been consistently associated with worse HF severity and outcomes in clinical investigations, although the strength of causal inference differs between designs. In a prospective cohort of 720 stable HF patients, greater plasma TMAO was related to increased long-term mortality, an effect that remained after adjustment for conventional risk variables, BNP, and renal function [60]. A second prospective study also demonstrated a rise in TMAO levels with NYHA functional class and an inverse association with transplant-free survival [61]. A meta-analysis of 6879 patients confirmed that higher TMAO was associated with increased major adverse cardiovascular events and all-cause mortality [62]; however, significant heterogeneity remained, even after adjustment for renal function, indicating that unmeasured confounding, inconsistent TMAO assay methodology or true population heterogeneity across the pooled studies limits how uniformly this association can be generalized.

Taken together, observations identify TMAO as a statistically significant predictive correlate of worse outcomes in HF, but not as a validated disease-specific biomarker or an established causative or therapeutic target. The main limitation, however, is that renal impairment, which is extremely common in HF and a major independent determinant of circulating TMAO, is difficult to fully separate from HF severity itself in observational designs, and persistent heterogeneity after renal adjustment suggests this confounding may not be fully resolved by statistical correction alone. Future prospective studies with mechanistic outcome data, e.g., whether microbiome-targeted reduction of TMA/TMAO production improves clinical endpoints independent of renal function, will be needed before the causative and therapeutic importance of TMAO in HF can be demonstrated.

#### 2.4.4. Atrial Fibrillation

The gut microbiota has been increasingly implicated in atrial fibrillation (AF), but the evidence base here is far less extensive than for atherosclerosis or heart failure—the literature comprises just a couple of cross-sectional studies rather than the larger, partly longitudinal evidence base available for those conditions, and no AF-specific mechanistic pathway has yet been experimentally validated. Changes in the structure of the gut microbial community in AF patients have been shown. One study comparing AF patients to non-AF controls described consistent patterns of dysbiosis in paroxysmal, persistent and permanent AF, suggesting that the association may be related to AF as a phenotype rather than to a specific clinical subtype [63]. However, as a cross-sectional comparison, this design cannot distinguish whether dysbiosis precedes the onset of AF or is a consequence of it—a distinction that matters considering that AF itself alters splanchnic perfusion and autonomic tone and that AF patients are often exposed to anticoagulants, antiarrhythmics and rate-control agents, any of which could independently remodel gut microbial composition.

Functional metagenomic data provide a more mechanistically specific, although still restricted, line of evidence. In one metagenomic comparison (n = 50 AF patients vs. 50 controls), AF patients were enriched for genes encoding enzymes involved in TMA-generating pathways, such as choline-TMA lyase, carnitine monooxygenase, and glycine betaine reductase [64]. This is the only metagenomic study of its kind in AF to date, and a single moderate-sized comparison is not sufficient to conclude that TMA-pathway enrichment is a reproducible or AF-specific feature as opposed to a chance finding or a correlate of the same comorbidities (obesity, diabetes, renal disease) discussed below.

Links between dysbiosis and atrial arrhythmogenesis have been proposed through several mechanisms, many of which are extrapolations of mechanisms described above in atherosclerosis and heart failure, including microbial modulation of systemic inflammation, autonomic tone, oxidative stress, endothelial function and atrial structural remodeling. Few, if any, of these pathways have been directly shown in atrial tissue or in AF-specific models; their inclusion here is based on biological plausibility rather than AF-specific mechanistic evidence. TMAO is the most common postulated potential connecting mediator, although a feasible pathway is not the same as a demonstrated pathway in this setting.

This evidence should be interpreted with special caution because AF is often accompanied by hypertension, obesity, diabetes, heart failure, coronary disease and chronic renal disease, each of which separately and dramatically changes the makeup of gut microbes. None of the studies reviewed above seems to fully isolate AF-specific microbiological signals from these shared comorbidities. The evidence in the present is therefore supportive of a relationship between gut dysbiosis and AF, but is not yet enough to determine a disease-specific microbial signature, a direct causative mechanism or even the direction of causality. Prospective, longitudinal studies are needed to determine if microbial change precedes AF onset and if targeting these pathways can reduce AF incidence or recurrence. These studies should be performed, if possible, in AF patients without major comorbid burden or with statistical adjustment for comorbidity and medication exposure.

#### 2.4.5. Integrative Perspective

Several recurrent gut microbiome–host cardiovascular physiology interactions can be observed across the cardiovascular disease spectrum. Atherosclerosis has been linked to altered microbial metabolic pathways, hypertension to changes in microbial diversity and SCFA-producing capacity, HF to intestinal barrier dysfunction and altered microbial metabolism, including TMAO-related pathways, and AF to dysbiosis and enrichment of TMA-producing pathways. These findings imply that cardiovascular phenotypes may be described by partially overlapping microbiome-related pathways, rather than by disease-specific microbial fingerprints.

This interpretation is particularly pertinent as atherosclerosis, hypertension, HF, and AF share several risk factors and pathophysiological processes, including systemic inflammation, endothelial dysfunction, metabolic abnormalities, thrombosis, and autonomic dysregulation. Thus, the gut microbiota may be one piece of a larger network connecting dietary intake, metabolism, immunology and cardiovascular physiology. Established cardiovascular illness can itself modify the intestinal environment through changes in perfusion, venous congestion, nutrition, drug exposure and host metabolism. Therefore, this association should be interpreted as a potential bidirectional rather than a linear chain of events in which dysbiosis occurs prior to cardiovascular disease.

Taken together, existing evidence indicates the existence of a gut–cardiovascular axis across many CVD symptoms but does not provide an overall disease-specific microbial signature, nor demonstrates that microbiome perturbations are independently causative. The obvious influence of diet, drugs, comorbidities, geography, age and host genetics complicates the challenge of identifying reproducible microbial biomarkers. Future investigations should thus go beyond taxonomic connections and include functional characterization of microbial pathways and metabolites, longitudinal designs that are able to establish temporal relationships and intervention trials with clinically significant cardiovascular endpoints. This technique will allow differentiation between microbiota changes that are causal mediators and those that are biomarkers or sequelae of established cardiovascular disease.

## 3. Therapeutic Modulation of the Gut–Cardiovascular Axis

The growing evidence suggests that the composition and function of the gut microbiota are linked to cardiovascular disease, leading to interest in therapies that target the gut–cardiovascular axis. These strategies include dietary manipulation, probiotic or prebiotic treatment, synbiotics and postbiotics, fecal microbiota transplantation (FMT), and targeted suppression of microbial metabolic pathways.

However, the data available varies considerably among interventions and clinical situations. Most studies have examined intermediate outcomes such as blood pressure, cholesterol concentrations, glycemic parameters, inflammatory markers, or microbiome composition rather than cardiovascular events. In addition, therapies modulating the microbiota may also affect many pathways in the host, making it difficult to establish if cardiovascular effects are particularly mediated via microbiome alterations. Direct comparison of research is also limited by heterogeneity in intervention composition, dose, duration, patient characteristics, baseline diet and microbiota, and analytical methodologies [65,66].

Accordingly, microbiome-directed therapies should be considered exploratory or supplementary techniques at this time and not substitutes for proven cardiovascular preventative and therapy strategies. Ultimately, their clinical relevance will depend on reproducible changes in microbial composition or metabolite profiles and on demonstrating clinically meaningful and persistent benefits in cardiovascular outcomes.

### 3.1. Dietary Modulation

Diet is one of the most accessible and potent factors of gut bacteria composition and metabolic activity. Dietary patterns with high intake of plant-derived foods, dietary fiber, resistant starch, polyphenols and unsaturated fatty acids may provide substrates that support microbial diversity and the production of potentially beneficial metabolites. Western-style dietary patterns characterized by high intakes of saturated fat, refined carbohydrates and ultra-processed foods have been associated with microbial alterations and metabolic disturbances. Among dietary patterns, the Mediterranean diet has gained particular interest as its cardioprotective effects could be mediated, at least in part, via interactions with the gut flora. Longitudinal analyses demonstrated that the link between the Mediterranean-style diet and lower cardiometabolic disease risk was mediated by gut microbiome makeup. Randomized dietary interventions can modify the microbial composition and profiles of fecal metabolites, and improve cardiometabolic risk factors [67,68]. But a key disadvantage is that cardiovascular benefits cannot usually be clearly linked to changes in the gut flora. Dietary patterns like the Mediterranean diet simultaneously modify calorie intake, fatty acid composition, exposure to fiber and polyphenols, glycemic load and countless other dietary parameters that independently affect cardiovascular risk. Therefore, even if a dietary intervention modifies the microbiome and improves cardiovascular risk factors, this does not indicate that the microbiome is the primary mediator of those benefits. Similarly, an increase in microbial diversity must not be seen as a therapeutic target per se. There is emerging evidence to support the assessment of specific diet–microbiome–host metabolic interactions rather than presuming that general changes in microbial composition will translate universally to cardiovascular benefit [47,69].

Dietary fiber may be of special importance because its fermentation by intestinal microbes generates short-chain fatty acids (SCFAs), such as acetate, propionate, and butyrate. These metabolites can affect intestinal barrier integrity, immunological responses, glucose and lipid metabolism, vascular function and blood pressure through interaction with G-protein-coupled receptors and other signaling pathways. Recent clinical evidence indicates that prebiotic fiber therapies can lower blood pressure in some hypertensive groups, with the magnitude of benefit dependent on substrate, dose, baseline microbiota, and host factors. Therefore, a dietary modulation should not be considered merely as an attempt to promote microbial diversity; rather, the functional ramifications of particular food–microbial interactions may be more important than taxonomic alterations per se [70,71].

Another route by which diet may alter the gut flora is via polyphenol-rich foods. Many dietary polyphenols undergo significant metabolism by the gut microbiota, resulting in metabolites with biological activity that can modulate inflammation, endothelial function, oxidative stress, and metabolic homeostasis. In turn, the microbiome can change the bioavailability and biological activity of dietary polyphenols, establishing a bidirectional interaction between dietary components and microbial metabolism. Dietary interventions may therefore affect microbial composition, microbial metabolic pathways and host signaling simultaneously, which could contribute to the cardiovascular advantages of plant-rich diets [72,73].

Importantly, the cardiovascular and microbiome-related effects of dietary interventions may also depend on the physicochemical properties of the food matrix delivering bioactive chemicals. Food structure and composition and processing conditions may alter the stability, release, bioaccessibility and subsequent intestine availability of dietary components [74,75,76,77]. These parameters could also impact the amount and chemical form of substrates arriving in the gut microbiota and, consequently, the diet–microbiome interactions [75]. For example, food processing may change the structure of plant tissues and the interactions between polyphenols and other food components, and thus their bioavailability and metabolism by the gut microbiota [76,77]. This means that the biological effects of a particular nutrient or bioactive chemical must not always be seen in isolation from the food matrix and processing circumstances. Therefore, in addition to nutrient composition and dosage, food structure, processing and matrix-dependent bioaccessibility should be additionally considered when evaluating the potential cardiovascular consequences of future microbiome-targeted dietary treatments.

### 3.2. Probiotics

Probiotics are live microorganisms that confer a health benefit on the host when administered in suitable doses. It is biologically possible that their potential cardiovascular relevance may entail modification of the intestinal microbial ecology, augmentation of epithelial barrier integrity, interaction with host immunological responses, and adjustment of microbial metabolite synthesis. These changes may then alter cholesterol and glucose metabolism, systemic inflammation and blood pressure. However, probiotic effects are very dependent on strain, dose, formulation, duration of administration and host. Therefore, data acquired with one microbe or formulation should not be directly extended to other probiotic preparations [78,79,80].

There is some evidence from human intervention trials to suggest that probiotics may alter selected cardiovascular risk indicators, although the amount and consistency of effects are variable. Meta-analyses of RCTs have demonstrated increases in parameters such as blood pressure, body weight, lipid concentrations and glycemic indices, but considerable heterogeneity has been detected between studies [81,82,83]. More subsequent investigations in subjects with coronary heart disease have also shown improvements in specific lipid markers [83]. Changes in surrogate markers of risk, however, should be evaluated with caution. Reduction in blood pressure and improvement in lipid concentrations or adjustment of glycemic indices alone do not demonstrate reduction in myocardial infarction, stroke, cardiovascular mortality or other clinical cardiovascular outcomes. The evidence so far gives a rationale for additional exploration but does not establish probiotics as an intervention that can prevent cardiovascular events.

Clinical findings may be inconsistent because of a variety of sources of heterogeneity. The term “probiotic” comprises a vast spectrum of bacterial and other microbial strains, combinations, doses, delivery matrices, and treatment schedules, each of which can have different biological effects. Baseline diet, drug use, metabolic profile, age and pre-existing microbiota may also influence the response to supplementation. Therefore, apparently conflicting results between trials should not be regarded as evidence that probiotics are essentially ineffective, but rather that the biological response is contingent on the strain–host interaction. This distinction is especially crucial to the interpretation of pooled analyses that mix mechanistically and clinically diverse preparations.

It is also unknown whether the introduced microorganisms have the ability to permanently alter the existing microbiota. Colonization and functional activity may be dependent on the makeup of the baseline microbiota of the receiver, availability of nutrients, circumstances of the intestinal environment, and concurrent medications. Hence, bacteria capable of exerting observable metabolic effects in one population may have quite different effects in a different population. Such environment dependence provides a reasonable explanation for the low repeatability of various microbiome and cardiometabolic outcomes between intervention studies [79,84].

Overall, the evidence does not support the use of probiotics as a uniform treatment class for cardiovascular protection. Therefore, future clinical trials should characterize the delivered strain or defined microbial mix, dose, viability, formulation and duration of treatment and should give priority to clinically relevant outcomes over surrogate markers alone. Baseline microbiome and metabolomic profiles may also help identify responders and non-responders and investigate whether unique host–microbe interactions account for variation reported across studies. Until such data are forthcoming, changes in the microbiome composition or intermediate markers of cardiovascular risk should be regarded as mechanistic or surrogate results, rather than direct evidence of cardiovascular benefit.

### 3.3. Prebiotics

Prebiotics are substrates that are selectively utilized by host bacteria that offer a health advantage [85]. They do not introduce live bacteria into the gastrointestinal tract as do probiotics, but rather adjust the availability of substrates for specific microbial populations and metabolic pathways. Examples from nutritional and cardiometabolic studies include inulin-type fructans, fructooligosaccharides, galactooligosaccharides and resistant starch. Their microbial fermentation can affect the formation of short-chain fatty acids and may additionally influence intestinal barrier function, glucose and lipid metabolism, inflammation and vascular physiology [85].

New clinical evidence indicates that microbiota-targeted treatments, such as prebiotics, may impact blood pressure, but the extent and reproducibility of the effect remain questionable. More direct evidence for this cardiovascular risk factor is provided by a systematic review and meta-analysis examining probiotics, postbiotics, prebiotics, and synbiotics specifically for hypertension [79]. However, these findings should be viewed as the wide variability of microbiome-targeted treatments and should not be regarded as evidence that all prebiotic formulations have similar antihypertensive effects.

One major cause of this heterogeneity is the fact that prebiotic responses depend on the features of the recipient microbiota. Experimental human data suggest that reactions to different prebiotics may be very consistent between individuals, but that individual traits may explain a large part of the heterogeneity regarding short-chain fatty acid responses [86]. Thus, habitual fiber consumption and baseline microbial features may alter the metabolic response to supplementation [86,87]. These data show that the action of a prebiotic cannot be appreciated solely from its chemical structure, but rather the substrate has to be examined in relation to the microbial population to which it is administered.

Such host–microbiome reliance has crucial consequences for clinical interpretation. Fermentable substrates can induce diverse microbial and metabolic responses, and the same substrate might elicit different responses in individuals with varied baseline microbiota. Thus, a positive discovery for one prebiotic preparation should not be readily extrapolated to prebiotics as a therapeutic class. Individualized responses to dietary fiber therapies [87] further reinforce the hypothesis that baseline microbiome composition and dietary exposure may contribute to responder and non-responder phenotypes.

Another restriction is that many of the studies are on intermediate cardiometabolic outcomes and over relatively short intervention periods. Even if changes are seen in blood pressure, lipid parameters, glucose metabolism or inflammatory markers, it is not clear whether these changes translate into long-term reductions in cardiovascular events. The current findings thus support the justification for further exploration of specific prebiotic therapies but not for a unified prebiotic-based strategy for cardiovascular prevention or treatment. Future trials should include characterization of the substrate delivered, as well as the baseline microbiota makeup, habitual dietary intake, dose, duration, and clinically relevant results.

### 3.4. Synbiotics

Synbiotics are defined as combination products of live microorganisms and substrates specifically used by host microorganisms, conferring a health advantage. Currently, it is generally accepted to differentiate between complementary synbiotics, where probiotic and substrate work separately, and synergistic synbiotics, when the substrate is especially designed to support the co-administered microbe [88]. This difference is clinically relevant as the biological rationale of a synbiotic rests not merely on the combination of two components but on the functional interaction between the microbe, substrate and host microbiota.

Several cardiometabolic risk factors have been studied in relation to synbiotics. The effect of probiotic and synbiotic formulations on outcomes such as body weight, waist circumference, lipid parameters, glycemic variables and blood pressure was investigated in a systematic review and meta-analysis of randomized controlled trials in healthy volunteers [89]. Some cardiometabolic risk factors were affected, but the authors also pointed out methodological limitations and varying quality of existing trials, which limit general conclusions [89]. More recent results on microbiota-targeted therapies and hypertension further support the importance of synbiotics to blood-pressure research and also point to the variety of formulations and study populations [79].

Synbiotic therapies are formulation-specific and thus create a major difficulty for evidence synthesis. Different products can contain different microbial strains, substrates, doses and treatment durations, and these components may interact differently with the recipient microbiome. So a positive result with one synbiotic combination cannot necessarily be extended to another preparation. Additionally, the expected interaction between the microbial and substrate components may not be consistent amongst individuals, as the baseline microbiome can affect substrate availability and microbial use [88].

These issues also apply to the evaluation of pooled clinical outcomes. The categorization of different synbiotic formulations as one intervention group may hide biologically important distinctions in strain characteristics, substrate consumption and mechanisms of action. Future trials should therefore include an extensive description of both components and test whether specific strain–substrate combinations lead to reproducible effects in defined populations.

The data now encourage additional research on the use of synbiotics in cardiometabolic disease, but are not yet sufficient to consider synbiotic treatment as a standardized cardiovascular intervention. To assess if improvements in intermediate risk indicators translate to cardiovascular benefit, well-designed randomized trials with well-described formulations, appropriate control groups, adequate follow-up and clinically significant outcomes are required [79,89].

### 3.5. Postbiotics

Postbiotics have evolved as an alternative to take advantage of microbial abilities without the administration of living microorganisms. The International Scientific Association for Probiotics and Prebiotics defines postbiotic as “a preparation of inanimate microorganisms and/or their components that confers a health benefit on the host” [90]. Depending on the preparation, postbiotics may comprise inactivated microbial cells, cell components, and other biologically active substances produced from microbes. The use of non-viable preparations may offer practical advantages over live microbial preparations in terms of stability, manufacture, storage and product handling, but these advantages are preparation-specific and should not be assumed for all postbiotics [90,91].

From a food technology perspective, the biological features of a postbiotic can be impacted by the source microorganism, the inactivation technique, the processing parameters, the formulation, the food matrix and the storage conditions [91,92]. Thus, inactivation is not only a method for removing microbial viability but can also influence the structural integrity, content and biological activity of microbial components. Interactions of postbiotic elements with the surrounding food matrix can also affect their stability, release and bioaccessibility in a similar way [91,92]. Therefore, while describing a product, the physicochemical stability and the conservation of the biological activity relevant to the intended use should be considered. Standardized reporting of the source microorganism, production and inactivation techniques, composition, significant bioactive components, and biological activity facilitates repeatability and meaningful comparison between experimental research [91,92].

The cardiovascular importance of postbiotics is mostly based on mechanistic research. Microbial components and metabolites can modulate pathways implicated in inflammation, intestinal barrier function, endothelial responses and metabolic balance [90,93]. However, molecular plausibility must be separated from documented cardiovascular efficacy. Clinical evidence, specifically pertaining to cardiovascular conditions, is still far behind the general experimental literature. The term “postbiotic” refers to preparations that may differ in composition and biological activities [90,91]. Therefore, an impact found for one defined postbiotic preparation cannot be automatically assumed to be the same for another preparation derived from another microorganism or created by another processing and inactivation approach.

This heterogeneity poses a major hurdle to evidence synthesis and therapeutic translation. Unlike traditional pharmacologic classes where chemicals may share a defined molecular target, postbiotic preparations may differ in microbial origin, cellular components, metabolites, production procedures and biological potency. Therefore, designation as a postbiotic should not be construed as proof of cardiovascular effectiveness per se. To establish a cardiovascular role, well-characterized formulations, reliable production methods, well-defined dosages and properly controlled human investigations would be needed [90,91,92,93].

Therefore, future research should combine standardized product characterization with mechanistic and clinical evaluation. In particular, it is not yet clear whether changes in inflammatory, metabolic, endothelial or other intermediate indicators are reproducible and whether these translate into clinically important cardiovascular outcomes. This differentiation is important, as biological activity exhibited in experimental models or surrogate indicators does not translate to clinical cardiovascular benefit.

### 3.6. Fecal Microbiota Transplantation

Fecal microbiota transplantation (FMT) is a general strategy to modulate the microbiome by transferring a complex microbial community from a screened donor to a recipient. While probiotics and prebiotics are usually directed at specific microbiota or microbiota functions, FMT may introduce a broader spectrum of microbiota and microbiota functions. This potential has attracted attention in metabolic disorders since changes in the intestinal microbiota have been correlated with obesity, insulin resistance, and cardiometabolic dysfunction [94].

However, existing evidence does not support FMT as an effective cardiovascular strategy. A systematic review and meta-analysis of FMT studies in cardiometabolic disorders evaluated outcomes including insulin sensitivity, lipid parameters, and microbiome-related changes, concluding that the available evidence was insufficient to demonstrate consistent clinically meaningful improvements across cardiometabolic outcomes [94]. A randomized controlled trial in individuals with metabolic syndrome and obesity provides further human data, but is limited by the study population, the intervention design and the duration of follow-up [95]. Thus, the evidence at the present time should be viewed as preliminary and not adequate to support normal cardiovascular application.

One of the major methodological issues is the diversity introduced by both donor and receiver characteristics. Donors might differ widely in microbial composition and functional capacity, and recipient parameters such as baseline microbiota, nutrition, metabolic status, immunological features and concomitant drugs may contribute to engraftment and clinical response [96]. The extent of microbial engraftment [96], together with donor-related, recipient-related and procedural parameters, determine therapeutic outcome in FMT studies. These observations quash the idea that a single definition of a “healthy donor” will be sufficient for all therapeutic reasons.

Another key factor is the persistence of microbiome alterations. Although transplantation can result in quantifiable changes in microbial composition, it is not yet clear whether these changes are lasting and whether persistent transfer is associated with clinically meaningful advantages. This is especially true for cardiovascular applications, where transient changes in microbiota or metabolic indicators may not translate into sustained changes in disease risk.

Another consideration is safety for clinical translation. Although FMT is an established treatment for recurrent *Clostridioides difficile* infection in appropriate clinical settings, its use in other indications demands careful assessment of potential transmission of infectious agents, antimicrobial-resistance determinants, and other undesirable biological characteristics. Additionally, the setting may influence donor screening, manufacturing techniques, routes of administration and regulatory requirements, which adds complexity to standardization [96].

The current evidence thus favors FMT as a microbiome-modulation method in cardiovascular research, rather than as a cardiovascular therapy. Future studies should include uniform donor selection and processing, robust safety monitoring, long-term follow-up and appropriately powered randomized designs. Also, of importance is whether certain donor or recipient microbiota traits might predict lasting clinical responses [94,95,96].

### 3.7. Microbial Enzyme Inhibitors

A more targeted approach to modulate microbiome-derived metabolic pathways is targeting microbial enzymes. One of the best studied examples is the trimethylamine (TMA)–trimethylamine-*N*-oxide (TMAO) pathway. Gut bacteria convert dietary substrates such as choline and carnitine to TMA, which is then oxidized to TMAO by host hepatic flavin-containing monooxygenases. This route has gained cardiovascular interest, as TMAO and associated microbial metabolism have been associated with atherosclerotic and thrombotic phenotypes [97,98]. However, the link between TMAO and cardiovascular disease does not prove that suppression of microbial TMA synthesis will lower cardiovascular events.

The microbial choline TMA-lyase encoded by the cutC/cutD system has therefore been explored as a possible therapeutic target. Preclinical investigations indicated that selective reduction of microbial TMA synthesis resulted in a decrease in circulating TMAO levels as well as an attenuation of atherosclerosis-related symptoms in experimental mice [99]. 3,3-dimethyl-1-butanol (DMB) was utilized as a non-lethal inhibitor of microbial TMA production and lowered TMAO levels and atherosclerosis-related alterations in mice, for example [99]. Other experimental inhibitors have been explored as potential strategies to target this system [97,100]. This strategy has the conceptual advantage of trying to change a specific microbial metabolic activity, without generally suppressing the gut microbial community.

However, the translational evidence remains weak, despite this mechanistic reason. Much of the experimental evidence has been obtained in preclinical models, and the extent to which targeted suppression of microbial TMA formation can safely induce clinically relevant cardiovascular consequences in humans remains uncertain [97,99]. Important considerations include the pharmacokinetics, long-term safety, implications on the broader microbial ecosystem, interactions with food, and whether chronic decreases in TMAO or related compounds translate into improved cardiovascular outcomes.

Another problem is the metabolic redundancy of the gut flora. Microbial routes for metabolite synthesis can be dispersed throughout different taxa and vary widely between people [97,100]. So, inhibition of one enzymatic pathway could be compensated for by alternative microbial processes or community structure changes. This potential places constraints on the assumption that alteration of one microbial enzyme would always yield a lasting therapeutic benefit.

Recent work on CutC strengthens the case for microbial choline metabolism as a viable therapeutic target, while underlining the importance of connecting molecular insight with translational development [100]. Future studies should integrate metagenomic and metabolomic characterization with targeted pharmacologic intervention to identify those in whom the relevant pathway is sufficiently active and clinically helpful. Critically, future therapy studies should distinguish between reductions in microbial metabolites or biomarkers and observed benefits in cardiovascular outcomes [97,100]. Inhibition of microbial enzymes is, at present, an exploratory strategy, with a good molecular basis, but inadequate clinical evidence to support its broad usage in cardiovascular therapy.

### 3.8. Therapeutic Perspective

Taken together, dietary treatments, probiotics, prebiotics, synbiotics, postbiotics, FMT, and microbial enzyme inhibitors provide many ways to modulate the gut–cardiovascular axis. They differ in microbiota specificity, reversibility, scalability, safety profile, and volume of clinical evidence.

Microbiome-targeted treatments vary widely in terms of specificity, clinical maturity, reversibility, scalability, safety, and strength of evidence. These discrepancies are relevant in relation to their possible involvement in cardiovascular medicine. Dietary modification offers the broadest clinical basis and the most potential for scaling, but we cannot attribute its cardiovascular benefits directly to microbiome changes since dietary interventions impact several host pathways simultaneously. Probiotics and prebiotics are reasonably easy to obtain and typically practical for long-term usage, but their effects are extremely reliant on strain or substrate properties, dose, host phenotype and baseline microbiome composition, and clinical outcomes have been variable. Synbiotics, a combination of microorganisms and defined substrates, may provide improved functional specificity; however, evidence is formulation-specific. While postbiotics might have certain benefits over live microbial preparations in terms of stability and safety, the variety of postbiotic products and the scant data on cardiovascular outcomes now limit their therapeutic interpretation.

However, the application of FMT in cardiovascular therapy is limited by donor variability, uncertainty in durability, concerns over infectious and other safety issues, challenges in standardization, and regulatory constraints. FMT may induce a more widespread and complicated modification in the microbiota than conventional supplementation. Targeted microbial enzyme inhibition offers a potentially more precise approach, as it aims to modulate a specific disease-associated microbial activity, not the microbiome in its entirety. However, most of these approaches are currently at a pre-clinical or early translational stage, and questions remain regarding pathway redundancy, inter-individual variance and long-term safety [65,66,94,100].

To summarize, the gut microbiome is increasingly becoming recognized not just as a biomarker of cardiovascular illness, but also as an actionable therapeutic target. But efficient translation will have to go from descriptive relationships to treatments on causally relevant microbial species, genes, pathways and metabolites. Overall, the available evidence does not support the concept that microbiome-directed therapies are interchangeable classes of therapy. The promise they hold for translation rests not only on their ability to alter microbial composition or activity but also on whether such changes translate to reproducible and clinically relevant cardiovascular benefit.

Future clinical trials should focus on well-described therapies, standardization of microbiome and metabolome measures, clinically significant cardiovascular outcomes and identification of responder phenotypes. Such precision-oriented approaches may ultimately allow microbiome modulation to become a complementary approach to standard cardiovascular prevention and treatment rather than a nonspecific therapeutic strategy [65,66,100].

## 4. Cardiovascular Drugs and the Microbiome

Most reviews on the gut microbiome–cardiovascular disease relationship have focused on the influence of the microbiome on the cardiovascular system, including the contributions of microbial dysbiosis and microbiota-derived metabolites to atherosclerosis, hypertension, thrombosis, heart failure, and other cardiovascular phenotypes. But this relationship is not one way. Cardiovascular medicines can themselves modulate the composition and metabolic activity of the gut microbiome, and the microbiome can modulate the pharmacokinetics and pharmacodynamics of cardiac treatments. This bidirectional link underlies the growing notion of drug–microbiome interactions or pharmacomicrobiomics as a potentially major predictor of cardiovascular therapy response [101,102,103].

The gut microbiome may affect drug disposition by many methods. Microbial enzymes may directly modify orally delivered medications before or after intestinal absorption, leading to the formation of metabolites with active, inactive or perhaps toxic properties. In addition, microbial metabolites can affect host drug-metabolizing enzymes and transporters, alter intestinal permeability and bile-acid metabolism, and modulate inflammatory and immunological pathways that are involved in pharmacological responses. Therefore, interindividual variation in microbial composition and function may contribute to variation in medication metabolism, bioavailability, efficacy and side effects [101,102,103]. Large-scale experimental analyses have demonstrated that gut bacteria can chemically modify a large fraction of commonly used drugs, offering a mechanistic basis for considering the microbiome as an additional contributor to pharmacological variability beyond host genetics [101,102].

### 4.1. Statins

Statins are one of the best studied examples of a potential bidirectional interaction between cardiovascular drugs and the gut microbiome. Statin drugs have a pharmacologic effect of blocking hepatic HMG-CoA reductase, but evidence is emerging that this is not a one-way interaction. Exposure to statins appears to remodel the gut microbiome, whereas baseline microbial composition may condition the lipid-lowering response [104,105,106]. However, the evidence base is strikingly inconsistent across research, and this is informative rather than incidental. Statin-induced microbial compositional shifts have been more consistently demonstrated in animal studies, whereas human data associating specific taxa to treatment response have not converged on the same bacterial signatures across cohorts, which is more consistent with population-specific confounding (diet, baseline dysbiosis, statin type and dose) than a single, generalizable microbial mechanism. This raises the question as to whether “the microbiome” is a proxy for unmeasured nutritional or metabolic variables rather than a causal, independent contributor.

The most consistent human signal to date associates a low-diversity microbiome profile enriched in *Bacteroides* with both increased lipid-lowering response and susceptibility to statin-associated metabolic side effects [105]—a finding that, if replicated, would be clinically significant precisely because it associates a single microbial signature with both efficacy and risk rather than with one or the other. But one-cohort findings of this sort are not yet able to differentiate a causal microbial mechanism from a common upstream cause, such as a host metabolic profile that modulates both microbiome composition and statin pharmacodynamics independently.

The bile-acid hypothesis offers a more mechanistically plausible link than composition-outcome correlations alone, since statins and bile acids share intestinal and hepatic transport pathways, and microbiota-dependent changes in the bile-acid pool could directly affect statin bioavailability rather than merely correlate with it [103,106]. This distinction is important because a shared-transporter mechanism is testable and falsifiable, whereas taxonomic association studies are not. Until pharmacokinetic studies precisely quantify statin exposure as a function of bile-acid-modifying microbial activity, the field has a plausible theory but not yet mechanistic proof. Microbiome-based stratification of statin medication is therefore a hypothesis-generating observation rather than a confirmed biomarker, and prospective studies designed to evaluate the mechanism—not only to reproduce associations—are the critical next step.

### 4.2. ACE Inhibitors and Angiotensin Receptor Blockers

The interaction between renin–angiotensin system (RAS) inhibitors and the gut microbiota is particularly interesting in relation to hypertension and cardiovascular remodeling, and here the directionality problem is very prominent. Angiotensin II has a direct impact on intestinal permeability, inflammation, and microbial composition. Thus, any change in the microbiome after initiation of an ACE inhibitor or ARB may be due to a direct drug–microbiome interaction, an indirect effect of lowering blood pressure and improving splanchnic perfusion, or a consequence of diet and lifestyle changes that often accompany initiation of antihypertensive therapy. The literature so far has seldom made any distinction between these options [107,108].

Experimental studies have linked RAS inhibition to enhanced microbial diversity and enrichment of SCFA-generating genera (*Bifidobacterium*, *Bacteroides*, *Blautia*, *Akkermansia)* and increases in acetate, propionate, and butyrate [107]. This is put out as proof that the medications change the microbiome in a good way. But the opposite causal path is equally consistent with the same data: SCFA-producing bacteria are known to expand under conditions of enhanced gut barrier integrity and reduced systemic inflammation, both downstream consequences of blood-pressure control rather than a specific pharmacological action on the microbiota. The published studies do not include the mediation analyses (e.g., assessing whether microbiome change precedes or follows blood-pressure change) would be needed to judge between these theories.

The fecal-transfer trials are the best evidence in this part because they are causal by design: transfer of microbiota from ARB-treated donors improved blood pressure, vascular remodeling, and oxidative stress in recipient animals [108]. This shows that the ARB-associated microbiota can provide some cardiovascular benefit in that model, a much more powerful argument than the correlational statin and human RAS-inhibitor results presented above. But translation is limited on two fronts, not one. The human evidence for RAS inhibitors altering the microbiome is itself minimal [107]. Even the animal causal evidence has not been taken further to ask whether ARB-attributable microbiome effects contribute a clinically meaningful fraction of antihypertensive efficacy in humans or are a minor epiphenomenon alongside a dominant direct pharmacological effect. This will require human trials with pre-treatment microbiome stratification and mediation modeling, not more descriptive taxonomic studies.

### 4.3. SGLT2 Inhibitors

Their cardiovascular benefit is already known to be greater than expected from glucose lowering alone, thus leaving open multiple possible mediators, natriuresis, metabolic remodeling, inflammation, oxidative stress and altered substrate use, of which the microbiome is only one, and not obviously the main one. Thus, SGLT2 inhibitors offer an attractive test case for the pharmacomicrobiomics framework.

The most robust single piece of evidence is the empagliflozin RCT, which is prospective in humans and associates microbiome remodeling (increased richness and diversity, enrichment of *Roseburia*, *Eubacterium*, and *Faecalibacterium*) with concurrent changes in circulating metabolites and cardiovascular risk parameters [109]. However, “linked to” is not “caused by”: improved glycemic control and decreased glucosuria-mediated caloric loss independently affect dietary intake, gut transit time, and luminal glucose availability, any of which could affect microbial composition without the microbiome itself contributing to the cardiovascular outcome. As described, the trial does not seem to have a mediation analysis that isolates the independent contribution of the microbiome from these co-occurring metabolic changes, so the microbiome remodeling finding, however robust, remains an association that runs in parallel to the cardiovascular benefit rather than a demonstrated causal contributor to it.

This caution is supported by the absence of consistency among other SGLT2 inhibitor studies rather than being noise: if microbiome remodeling were a major mediator of a class-wide cardiovascular effect, one would expect more convergent microbial signatures across agents with a common mechanism of action. The finding that different SGLT2 inhibitors, treatment durations, and dietary contexts provide diverse microbial results is more consistent with the microbiome acting as a downstream marker of differential metabolic response rather than an upstream driver of therapeutic efficacy. Therefore, current evidence supports an association between SGLT2 inhibition and microbiome remodeling, but the causal architecture (whether the microbiome contributes to, is a consequence of, or is coincident with the cardiovascular effects of the drugs) remains unresolved.

### 4.4. Aspirin

The preclinical data on the bile-acid-repair axis are causal by design, as an experimental model showed that microbiota-dependent bile-acid signaling is involved in intestinal repair following aspirin-induced damage [110], directly supporting the claim that the microbiome is an active participant, rather than a passive victim, of aspirin’s gastrointestinal effects. This is a mechanistically informative observation, as it points to a specific mechanism (bile-acid signaling) rather than an unspecific “microbiome effect”.

The human observational data are of a different evidential nature. Long-term use of aspirin has been linked to altered gut microbial composition, particularly when used in combination with atorvastatin [111]. However, these cohorts are typically characterized by polypharmacy and heterogeneous cardiovascular risk profiles, making it impossible to separate the aspirin-specific effect—the observation is consistent with aspirin causing the alteration, atorvastatin causing it, the combination causing it synergistically, or the underlying common cardiovascular risk profile (diet, comorbidity burden) driving both drug exposure and microbiome state independently. Without aspirin-monotherapy cohorts or dose-stratified analyses, this observational finding cannot be utilized to support a specific aspirin-microbiome mechanism, merely to motivate one.

The synthesis of these two types of evidence yields an asymmetrical picture: there is a plausible, mechanistically demonstrated pathway whereby the microbiome is involved in repair from aspirin-induced GI injury, but there are, as yet, no data of comparable rigor that establish the baseline microbiome composition that can predict susceptibility to aspirin-related GI harm in humans. Neither strand of current evidence answers the clinically relevant question of whether microbiome profiling can help identify patients at increased risk of aspirin-related GI bleeding while retaining its cardiovascular benefit. This question should be posed as the specific and testable knowledge gap it is, rather than a general call for “further studies”.

### 4.5. Antiplatelet Drugs

The primary assertion, that TMAO modifies platelet reactivity and hence may affect the biological milieu in which antiplatelet agents operate [112], is a pharmacodynamic hypothesis rather than an established interaction. It has biological plausibility (TMAO impacts platelets; the microbiota creates TMAO), but no proof has been made that levels of TMAO obtained from the microbiome truly predict antiplatelet medication efficacy, resistance, or bleeding risk in patients.

The clopidogrel data do not resolve this problem; they add to it. High on-treatment platelet reactivity has been associated with metabolic abnormalities, including obesity and heart failure, as well as insulin resistance, which is independently connected with gut dysbiosis [113]. This is a classic confounder scheme where the metabolic disorder could influence both platelet reactivity and microbiota composition through common pathways (systemic inflammation, altered bile acid and lipid metabolism) without either variable being the cause of the other. Using this connection as evidence of the microbiome-antiplatelet-response link without considering the shared-confounder explanation exaggerates the evidence in the data.

The evidence we have now supports a biologically plausible hypothesis—that microbial metabolites could affect the platelet-vascular environment in which antiplatelet medications work—but not a confirmed clinical benefit. In essence, there is no direct evidence relating distinct microbial taxa or metabolite profiles to measurable differences in antiplatelet efficacy or bleeding outcomes (Summary in Table 2).

### 4.6. From Microbiome–CVD Associations to Pharmacomicrobiomics

Taken together, the data indicates a paradigm shift in the way the gut microbiome is perceived in cardiovascular care. The conventional paradigm suggests that changes in the composition of microbiomes contribute to cardiovascular disease through microbial metabolites, compromised intestinal barrier function, inflammation, and metabolic dysregulation. A more complete model must also account for the fact that cardiovascular medication can remodel the microbiome and the microbiota can modify drug disposition and response.

Hence, cardiovascular medications and gut microbes may represent a dynamic bidirectional system:

Cardiovascular drug → gut microbiome → microbial metabolites/enzymes → host metabolism and signaling → drug efficacy and adverse effects.

With a reciprocal pathway:

Gut microbiome → drug metabolism/bioavailability → pharmacological exposure → cardiovascular response.

This paradigm could serve to explain part of the large interindividual heterogeneity in response to cardiovascular medication. Two patients treated with the same medication at the same dose may have different microbial communities, variable microbial metabolic capabilities and therefore possibly varied drug exposure, therapeutic responses or susceptibility to adverse effects. Importantly, this hypothesis does not suggest that microbiome differences are currently ready for clinical use, but suggests an additional potential level of biological diversity that warrants future exploration.

The emerging field of pharmacomicrobiomics thereby offers an opportunity to move beyond the conventional question of whether the microbiome contributes to cardiovascular disease, and toward a broader question: how does the microbiome influence the effectiveness and safety of cardiovascular treatment, and how do cardiovascular drugs in turn reshape the microbial ecosystem? Answering this question may lead to a more customized cardiovascular medication in which microbial composition and activity are evaluated along with clinical features, host genetics, diet and conventional pharmacokinetic and pharmacodynamic determinants [101,102,103].

## 5. Challenges and Future Perspectives

Although evidence for the function of the gut microbiome in CVD is accumulating, there are still several critical issues that need to be addressed before microbiome-targeted strategies may be included in routine cardiovascular prevention and therapy. The present database is characterized by significant variation in study populations, illness phenotypes, food patterns, medication exposure, microbiome gathering and sequencing techniques, analytical pipelines, and clinical outcomes. Consequently, apparently identical studies might find different microbial profiles or report conflicting relationships with cardiovascular risk. These recent initiatives to standardize reporting of clinical information, sampling protocols, analytical approaches and clinical objectives highlight that harmonization will be critical to enhance reproducibility and comparability in microbiome research [114,115].

Another problem is the tendency to look at microbiome changes at the genus or species level, despite large functional differences between individual strains. Close relatives of microbial strains may differ greatly in their metabolic capacities, host interactions and effect on inflammation or cardiovascular risk. Thus, the classification of a bacterial genus as “beneficial” or “harmful” may be an inadequate description of its biological consequences. Future research should combine strain-level resolution with functional profiling, metagenomics, metatranscriptomics and metabolomics to understand which microbial activities, rather than which taxa, are linked to cardiovascular abnormalities. This distinction is especially essential for probiotic research as therapeutic effects are often strain-specific and cannot necessarily be generalized from one bacterium to another [116,117].

Another key challenge to the development of universally effective microbiome-based medicines is the heterogeneity across individuals. The composition and functional capacity of the gut microbiome are modified by age, genetics, diet, lifestyle, geography, exposure to medicine, metabolic status, and other environmental factors. Importantly, individuals with comparable clinical characteristics may have quite varied microbiome compositions and may respond differentially to the same intervention. The variability is the rationale for moving away from a one-size-fits-all approach toward precision microbiome medicine, where baseline microbial composition and metabolic characteristics could be taken into account when choosing dietary, probiotic, prebiotic, postbiotic or metabolite-directed interventions. Eventually, this method could enable the combination of microbiome-based approaches with classical risk factors and other molecular characteristics in cardiovascular medicine to define individuals who are most likely to benefit from a particular intervention [117,118].

Therefore, the creation of reliable microbiome-derived biomarkers is a highly significant direction for the future. Potential to aid in cardiovascular risk stratification, early disease diagnosis, prediction of disease progression, or identification of therapy responders includes microbial taxa, microbial genes, metabolic pathways, and circulating microbial metabolites. However, many of the proposed biomarkers are derived from observational research and could be surrogate markers for disease-associated alterations rather than causal factors. Prospective validation in independent and heterogeneous populations, assessment of incremental predictive value beyond established cardiovascular risk factors, and confirmation that biomarker-guided decisions enhance patient outcomes will be required for their therapeutic relevance. The integration of microbiome data with other omics data, including metabolomics, proteomics, genomics and clinical data, is discussed for its potential significance since the composition of microbiota alone may not be enough to reflect the functional status of the gut environment [114,119].

Such advances may enable the creation of tailored probiotics and next-generation microbiome therapies. Traditionally, probiotics have been administered using a population-based strategy, with a single strain or mix of strains given to subjects with varied baseline microbiomes. A precision approach, however, would instead use pre-treatment microbiome profiling and functional characterization to choose strains or microbial consortia with the highest probability of colonizing, functioning, or producing the intended metabolic effect in a specific host. Recent work has suggested the promise of combining multi-omics profiling, predictive modeling and strain engineering to generate more focused probiotic therapies. However, tailored probiotics are an emerging idea and many critical questions concerning efficacy, long-term safety, strain selection, manufacturing uniformity, regulatory classification and clinical validation are still to be answered [120,121].

Another interesting breakthrough is the application of artificial intelligence (AI) and machine learning to the increasingly complex data sets generated by microbiome research. Conventional statistical approaches may not be sufficient to capture non-linear connections among microbial taxa, microbial metabolites, host features, food, pharmaceuticals and cardiovascular phenotypes. AI-assisted techniques might combine metagenomic, metabolomic, proteomic, clinical, and lifestyle data to discover complex microbial signatures, find candidate biomarkers, predict individual responses to microbiome-targeted treatments, and potentially aid therapy selection. Recent reviews have pointed to the potential of AI and integration of multi-omics for biomarker discovery and prediction of therapy response, but also highlighted that model interpretability, data quality, external validation and clinical implementation are still key challenges [119,122].

Critically, computer prediction should not be taken as a replacement for biological validation. Associations revealed by machine-learning models need to be validated by experimental methodologies that can show causation, including mechanistic in vitro systems, animal models, organoids and well-planned human intervention trials. Consensus efforts in recent years have explicitly addressed the significance of strengthening preclinical models and standardizing experimental procedures to improve causal inference and translational reproducibility in microbiome research [123]. Likewise, AI models trained on data from one community may not perform well in another due to changes in nutrition, ethnicity, geography, drug use, sequencing platforms, or illness prevalence. Therefore, future research on microbiome-AI ought to focus on large, diversified, and longitudinal datasets and thorough external validation rather than strong prediction performance within single cohorts.

In the end, for the translation of microbiome research to cardiovascular treatment to be successful, it will be necessary to move beyond descriptive relationships to interventions that are mechanistically informed, reproducible and clinically actionable. To identify causative microbiome abnormalities that can be therapeutically targeted, standardized methodology, strain-level characterization, longitudinal sampling, integration of multi-omics data, and well-designed randomized clinical trials will be necessary. Thus, the field is ready to transition beyond the identification of particular “beneficial” or “harmful” bacteria, to an understanding of individualized microbial roles and host–microbe interactions. In this respect, the future of microbiome-targeted cardiovascular medicine might not be a single universal probiotic or microbial target but rather an individualized therapeutic framework encompassing the patient’s microbiome, microbial metabolites, diet, medications, clinical phenotype and other molecular characteristics. Such a strategy could eventually transform the gut microbiota from a mostly observational measure of cardiovascular health into a therapeutically useful component of precision cardiovascular care [114,118,119,122].

## 6. Conclusions

Diet, metabolism, immunity, and cardiovascular health are linked by a complex biological network where the gut microbiota has emerged as a crucial player. Emerging data indicates that changes in microbial makeup and activity are linked to major cardiovascular diseases, such as atherosclerosis, hypertension, heart failure, and thrombotic illness. Microbial-derived metabolites, including trimethylamine-*N*-oxide, short-chain fatty acids, bile acids and indole derivatives, are important mechanistic links between intestinal microbial activity and cardiovascular physiology, albeit with highly context-dependent effects that cannot simply be reduced to a distinction between “beneficial” and “harmful” metabolites.

From a nutritional point of view, the therapeutic potential of addressing the gut microbiome is particularly relevant. Dietary patterns, dietary fiber, polyphenols, prebiotics, probiotics, synbiotics and postbiotics may alter cardiovascular risk by their impact on microbial composition and metabolic activity. Other techniques include fecal microbiota transplantation and targeted suppression of certain microbial metabolic pathways, although their use in cardiovascular disease remains experimental. Importantly, microbiome-directed therapies should be viewed as complimentary to known cardiovascular preventative and treatment measures and not as substitutes for evidence-based pharmaceutical therapy.

An important and developing part of the gut–heart axis is the bidirectional interaction with cardiovascular drugs. Statins, renin–angiotensin system inhibitors, SGLT2 inhibitors, aspirin and anti-platelet treatments may modulate the intestinal microbiome and microbial enzymes and metabolites may influence medication metabolism, pharmacological efficacy and side effects. This new science of pharmacomicrobiomics opens up the possibility that interindividual differences in the microbiome may be an additional factor contributing to interindividual differences in cardiovascular therapy response, in addition to genetic, metabolic, nutritional, and environmental factors.

While significant progress has been made, some problems are now hampering translation into clinical practice. Differences in research populations, nutritional intake, medication usage, microbiome profiling techniques, sequencing tactics and analytical tools make studies difficult to compare. Furthermore, relationships between particular taxa and cardiovascular symptoms are not always causal, and the functional capacity of the microbiome may be more revealing than taxonomic makeup alone. Future research should therefore go beyond descriptive characterization to longitudinal, mechanistic and sufficiently powered clinical studies incorporating metagenomics, metabolomics, host genetics, dietary information and clinical phenotyping.

Among the currently available microbiome-targeted strategies, dietary interventions, especially healthy diets rich in fiber and polyphenols, seem to have the most translational potential, due to their broader metabolic and cardiovascular effects and established role in cardiovascular prevention. Prebiotics, probiotics, synbiotics and postbiotics are promising, but more context-dependent treatments where clinical efficacy is strain- or formulation-specific, host-specific, depending on baseline microbiota and impacted by dietary backdrop. Fecal microbiota transplantation and targeted inhibition or regulation of certain microbial metabolic pathways are highly experimental in the setting of cardiovascular disease and require considerably more information as to efficacy, safety, patient selection, and long-term effects.

However, several mechanistic gaps need to be addressed before microbiome-based therapies may be implemented in precision cardiovascular therapy. These include determining causal links between specific microbial functions and cardiovascular events, whether microbial metabolites or taxa are appropriate therapeutic targets, how individuals differ in their response to treatment, and identifying robust biomarkers that can predict clinical benefit. Future investigations should thus aim at long-term well-powered clinical trials that include metagenomics, metabolomics, host genetics, dietary data, pharmaceutical exposure and thorough cardiovascular phenotyping. Such research will be key to assessing whether the microbiota can be manipulated, and whether such manipulation can consistently result in clinically substantial changes in cardiovascular outcomes.

## Figures and Tables

**Table 1:** Major gut microbiota-derived mechanisms linking intestinal dysbiosis to cardiovascular disease and their potential therapeutic implications.

Gut Microbial Pathway/Metabolite	Major Microbial or Host Processes	Principal Mechanisms Relevant to CVD	Potential Cardiovascular Consequences	Potential Therapeutic Implications
**Trimethylamine–TMAO pathway**	Microbial conversion of choline, phosphatidylcholine, and L-carnitine to trimethylamine (TMA), followed by hepatic oxidation to TMAO by flavin-containing monooxygenases (FMO3)	Altered cholesterol metabolism; impaired reverse cholesterol transport; vascular inflammation; endothelial dysfunction; increased platelet reactivity and thrombosis	Atherosclerosis, coronary artery disease, increased thrombotic risk	Dietary substrate modification; modulation of TMA-producing bacteria; inhibition of microbial TMA formation; modulation of host TMAO production
**Short-chain fatty acids (SCFAs)**	Microbial fermentation of dietary fiber and resistant carbohydrates produces acetate, propionate, and butyrate	Activation of FFAR2/GPR43 and FFAR3/GPR41; histone deacetylase inhibition; immune regulation; maintenance of intestinal barrier integrity; modulation of vascular and metabolic signaling	Potential protection against inflammation, hypertension, endothelial dysfunction, metabolic disturbances	Increased fermentable dietary fiber; prebiotics; probiotics; next-generation microbial therapies; enhancement of SCFA-producing pathways
**Bile acid–microbiota axis**	Microbial deconjugation and transformation of primary bile acids into secondary bile acids	FXR and TGR5 signaling modulation; regulation of lipid and glucose metabolism; inflammatory control; cholesterol homeostasis	Altered cardiometabolic risk; contribution to atherosclerosis via bile acid signaling imbalance	Dietary modulation; microbiota-targeted interventions; bile acid receptor agonists/antagonists; modulation of microbial bile acid metabolism
**Intestinal barrier dysfunction and metabolic endotoxemia**	Dysbiosis-induced disruption of mucus layer, epithelial integrity, and tight junctions; increased translocation of microbial products (e.g., LPS)	LPS/TLR4 activation; NF-κB signaling; systemic inflammation; oxidative stress; endothelial dysfunction; metabolic impairment	Chronic inflammation, insulin resistance, hypertension, endothelial dysfunction, vascular disease	Restoration of gut barrier integrity; dietary interventions; microbiota modulation; reduction of pro-inflammatory microbial products
**Phenylacetylglutamine (PAGln)**	Microbial metabolism of phenylalanine to phenylacetic acid followed by host conjugation with glutamine	Activation of adrenergic receptors; increased platelet reactivity; pro-thrombotic signaling	Increased thrombosis risk and cardiovascular events	Inhibition of PAGln-producing microbial pathways; modulation of precursor availability; microbiota-targeted interventions
**Hydrogen sulfide (H_2_S)**	Production by host and sulfur-metabolizing gut microbes from sulfur-containing substrates	Regulation of vascular tone; oxidative stress modulation; anti-inflammatory effects; smooth muscle and platelet regulation	Effects on blood pressure regulation, vascular homeostasis, atherosclerosis (dose-dependent)	Modulation of sulfur-metabolizing microbiota; controlled H_2_S donor strategies (experimental)
**Branched-chain amino acids (BCAAs)**	Metabolism of leucine, isoleucine, and valine influenced by diet, host metabolism, and microbial activity	Altered nutrient sensing; mitochondrial dysfunction; insulin resistance; metabolic stress; vascular effects	Association with cardiometabolic disease and incident CVD	Dietary modification; metabolic improvement; modulation of BCAA-related microbial pathways

Abbreviations: AhR, aryl hydrocarbon receptor; BCAAs, branched-chain amino acids; CVD, cardiovascular disease; FFAR2, free fatty acid receptor 2; FFAR3, free fatty acid receptor 3; FMO3, flavin-containing monooxygenase 3; FXR, farnesoid X receptor; H_2_S, hydrogen sulfide; LPS, lipopolysaccharide; NF-κB, nuclear factor-κB; PAGln, phenylacetylglutamine; SCFAs, short-chain fatty acids; TMA, trimethylamine; TMAO, trimethylamine *N*-oxide; TGR5, Takeda G-protein-coupled bile acid receptor.

**Table 2:** Bidirectional interactions between cardiovascular medications and the gut microbiome.

Cardiovascular Drug/Class	Effects on the Gut Microbiota	Proposed Microbiome-Mediated Mechanisms	Potential Clinical Implications
Statins	Statin treatment may modify microbial composition, diversity, and metabolic activity. Conversely, baseline microbiome composition has been associated with variability in statin response and metabolic adverse effects.	Microbiota-dependent bile acid metabolism may influence cholesterol homeostasis and host signaling through FXR/TGR5 pathways. Microbial composition may also affect drug metabolism and systemic metabolic responses.	May contribute to interindividual variability in LDL-C lowering and susceptibility to adverse metabolic effects. Gut microbiome profiles may eventually serve as biomarkers of statin response.
ACE inhibitors	Experimental studies suggest alterations in microbial diversity and enrichment of taxa potentially associated with SCFA production following RAS inhibition.	Microbial SCFAs may influence vascular tone, immune signaling, intestinal barrier integrity, and blood-pressure regulation.	Gut microbiome modulation may contribute to some pleiotropic effects of ACE inhibition, although clinical relevance remains uncertain.
Angiotensin receptor blockers (ARBs)	ARB treatment has been associated experimentally with changes in *Bifidobacterium*, *Bacteroides*, *Blautia*, *Akkermansia*, and microbial metabolites.	Changes in microbial metabolites, particularly SCFAs, may affect vascular inflammation, oxidative stress, intestinal integrity, and blood-pressure regulation.	Microbiome remodeling may contribute to antihypertensive and vascular-protective effects. Evidence remains predominantly preclinical.
SGLTS2 inhibitors	Empagliflozin has been associated with increased microbial richness and diversity and enrichment of SCFA-producing genera, including *Roseburia*, *Eubacterium*, and *Faecalibacterium*.	Microbiome changes may influence circulating metabolites, inflammation, energy metabolism, and cardiometabolic signaling.	Microbiome remodeling may contribute to the cardiovascular benefits of SGLT2 inhibitors beyond glucose lowering. Causality remains to be established.
Aspirin	Aspirin may alter gut microbial activity and microbial–bile acid interactions, particularly following prolonged exposure.	Microbiota-dependent bile acid signaling may influence epithelial repair, intestinal barrier function, and inflammatory responses following aspirin-induced injury.	Individual microbiome characteristics may contribute to differences in gastrointestinal tolerance and susceptibility to aspirin-associated mucosal injury.
Clopidogrel	Direct effects of clopidogrel on the microbiome remain insufficiently characterized. Microbial metabolic capacity may nevertheless contribute to variability in oral drug disposition.	Microbial metabolic pathways and host metabolic status may influence drug exposure and platelet response. Microbial metabolites such as TMAO may additionally modulate platelet reactivity.	May contribute to interindividual variability in antiplatelet response and thrombotic risk, although direct causal links remain limited.
Antiplatelet therapy—microbial metabolite interaction	Microbial metabolites can influence platelet biology independently of direct drug metabolism.	TMAO can enhance platelet hyperreactivity and thrombosis through effects on platelet signaling and vascular biology.	The microbiome may modify the biological environment in which antiplatelet drugs act and could potentially influence the balance between thrombotic protection and bleeding risk.

Abbreviations: ACE, angiotensin-converting enzyme; ARB, angiotensin receptor blocker; FXR, farnesoid X receptor; LDL-C, low-density lipoprotein cholesterol; RAS, renin–angiotensin system; SCFA, short-chain fatty acid; SGLT2, sodium–glucose cotransporter 2; TGR5, Takeda G-protein-coupled bile acid receptor 1; TMAO, trimethylamine-*N*-oxide.

## Data Availability

No new data were created or analyzed in this study. Data Sharing is not applicable to this article.

## Abbreviations

The following abbreviations are used in this manuscript:

CVD	cardiovascular disease
ASCVD	atherosclerotic cardiovascular disease
BCAA	branched-chain amino acids
GI	gastrointestinal tract
ACE	angiotensin-converting enzyme
ARB	angiotensin receptor blocker
FXR	farnesoid X receptor
LDL-C	low-density lipoprotein cholesterol
RAS	renin–angiotensin system
SCFA	short-chain fatty acid
SGLT2	sodium–glucose cotransporter 2
TGR5	Takeda G-protein-coupled bile acid receptor 1
TMAO	trimethylamine-*N*-oxide
AhR	aryl hydrocarbon receptor
BCAAs	branched-chain amino acids
FFAR2	free fatty acid receptor 2
FFAR3	free fatty acid receptor 3
FMO3	flavin-containing monooxygenase 3
H_2_S	hydrogen sulfide
LPS	lipopolysaccharide
NF-κB	nuclear factor-κB
PAGln	phenylacetylglutamine
GPR43/41	G-protein-coupled receptor 43/41
TLR4	toll-like receptor

## References

[1] World Health Organization (2025). Cardiovascular Diseases (CVDs).

[2] Visseren F.L.J., Mach F., Smulders Y.M., Carballo D., Koskinas K.C., Bäck M., Benetos A., Biffi A., Boavida J.M., Capodanno D. (2021). 2021 ESC Guidelines on cardiovascular disease prevention in clinical practice. Eur. Heart J..

[3] Wang L., Wang S., Zhang Q., He C., Fu C., Wei Q. (2022). The role of the gut microbiota in health and cardiovascular diseases. Mol. Biomed..

[4] Zaher A., Elsaygh J., Peterson S.J., Weisberg I.S., Parikh M.A., Frishman W.H. (2026). The interplay of microbiome dysbiosis and cardiovascular disease. Cardiol. Rev..

[5] Thursby E., Juge N. (2017). Introduction to the human gut microbiota. Biochem. J..

[6] Evans J.M., Morris L.S., Marchesi J.R. (2013). The gut microbiome: The role of a virtual organ in the endocrinology of the host. J. Endocrinol..

[7] Fan Y., Pedersen O. (2021). Gut microbiota in human metabolic health and disease. Nat. Rev. Microbiol..

[8] Usman I., Anwar A., Shukla S., Pathak P. (2024). Mechanistic Review on the Role of Gut Microbiota in the Pathology of Cardiovascular Diseases. Cardiovasc. Hematol. Disord. Drug Targets.

[9] Lin L., Xiang S., Chen Y., Liu Y., Shen D., Yu X., Wu Z., Sun Y., Chen K., Luo J. (2024). Gut microbiota: Implications in pathogenesis and therapy to cardiovascular disease (Review). Exp. Ther. Med..

[10] Tang W.H.W., Bäckhed F., Landmesser U., Hazen S.L. (2019). Intestinal Microbiota in Cardiovascular Health and Disease: JACC State-of-the-Art Review. J. Am. Coll. Cardiol..

[11] Li C., Stražar M., Mohamed A.M.T., Pacheco J.A., Walker R.L., Lebar T., Zhao S., Lockart J., Dame A., Thurimella K. (2024). Gut microbiome and metabolome profiling in Framingham heart study reveals cholesterol-metabolizing bacteria. Cell.

[12] Martins D., Silva C., Ferreira A.C., Dourado S., Albuquerque A., Saraiva F., Batista A.B., Castro P., Leite-Moreira A., Barros A.S. (2024). Unravelling the Gut Microbiome Role in Cardiovascular Disease: A Systematic Review and a Meta-Analysis. Biomolecules.

[13] Wang Z., Klipfell E., Bennett B.J., Koeth R., Levison B.S., Dugar B., Feldstein A.E., Britt E.B., Fu X., Chung Y.M. (2011). Gut flora metabolism of phosphatidylcholine promotes cardiovascular disease. Nature.

[14] Koeth R.A., Wang Z., Levison B.S., Buffa J.A., Org E., Sheehy B.T., Britt E.B., Fu X., Wu Y., Li L. (2013). Intestinal microbiota metabolism of L-carnitine, a nutrient in red meat, promotes atherosclerosis. Nat. Med..

[15] Mukhopadhya I., Louis P. (2025). Gut microbiota-derived short-chain fatty acids and their role in human health and disease. Nat. Rev. Microbiol..

[16] Fleishman J.S., Kumar S. (2024). Bile acid metabolism and signaling in health and disease: Molecular mechanisms and therapeutic targets. Signal Transduct. Target. Ther..

[17] Chen F., Gong L. (2025). Bile acid–microbiota interactions in cardiometabolic diseases: Mechanisms and emerging therapeutic approaches. Front. Microbiol..

[18] Cani P.D., Amar J., Iglesias M.A., Poggi M., Knauf C., Bastelica D., Neyrinck A.M., Fava F., Tuohy K.M., Chabo C. (2007). Metabolic endotoxemia initiates obesity and insulin resistance. Diabetes.

[19] Chen B., Gautron L. (2025). Gut-derived lipopolysaccharides and metabolic endotoxemia: A critical review. Am. J. Physiol. Endocrinol. Metab..

[20] Yang T., Maki K.A., Marques F.Z., Cai J., Joe B., Pepine C.J., Pluznick J.L., Meyer K.A., Kirabo A., Bennett B.J. (2025). Hypertension and the Gut Microbiome: A Science Advisory From the American Heart Association. Hypertension.

[21] Shoukry A.E.A., Rahhal A., Constantinou C. (2025). The role of the gut microbiota and metabolites in heart failure and possible implications for treatment. Heart Fail. Rev..

[22] Khuu M.P., Paeslack N., Dremova O., Benakis C., Kiouptsi K., Reinhardt C. (2025). The gut microbiota in thrombosis. Nat. Rev. Cardiol..

[23] Human Microbiome Project Consortium (2012). Structure, function and diversity of the healthy human microbiome. Nature.

[24] Hou K., Wu Z.X., Chen X.Y., Wang J.Q., Zhang D., Xiao C., Zhu D., Koya J.B., Wei L., Li J. (2022). Microbiota in health and diseases. Signal Transduct. Target. Ther..

[25] Camilleri M. (2019). Leaky gut: Mechanisms, measurement and clinical implications in humans. Gut.

[26] Chiang J.Y. (2013). Bile acid metabolism and signaling. Compr. Physiol..

[27] Levy M., Kolodziejczyk A.A., Thaiss C.A., Elinav E. (2017). Dysbiosis and the immune system. Nat. Rev. Immunol..

[28] Belkaid Y., Hand T.W. (2014). Role of the microbiota in immunity and inflammation. Cell.

[29] Kriss M., Hazleton K.Z., Nusbacher N.M., Martin C.G., Lozupone C.A. (2018). Low diversity gut microbiota dysbiosis: Drivers, functional implications and recovery. Curr. Opin. Microbiol..

[30] Petersen C., Round J.L. (2014). Defining dysbiosis and its influence on host immunity and disease. Cell. Microbiol..

[31] Shen Y., Fan N., Ma S.-X., Cheng X., Yang X., Wang G. (2025). Gut Microbiota Dysbiosis: Pathogenesis, Diseases, Prevention, and Therapy. MedComm.

[32] Acevedo-Román A., Pagán-Zayas N., Velázquez-Rivera L.I., Torres-Ventura A.C., Godoy-Vitorino F. (2024). Insights into Gut Dysbiosis: Inflammatory Diseases, Obesity, and Restoration Approaches. Int. J. Mol. Sci..

[33] Safarchi A., Al-Qadami G., Tran C.D., Conlon M. (2025). Understanding Dysbiosis and Resilience in the Human Gut Microbiome: Biomarkers, Interventions, and Challenges. Front. Microbiol..

[34] Clemente-Suárez V.J., Beltrán-Velasco A.I., Redondo-Flórez L., Martín-Rodríguez A., Tornero-Aguilera J.F. (2023). Global Impacts of Western Diet and Its Effects on Metabolism and Health: A Narrative Review. Nutrients.

[35] Cheng B., Feng H., Li C., Jia F., Zhang X. (2025). The Mutual Effect of Dietary Fiber and Polyphenol on Gut Microbiota: Implications for the Metabolic and Microbial Modulation and Associated Health Benefits. Carbohydr. Polym..

[36] Du Y., He C., An Y., Huang Y., Zhang H., Fu W., Wang M., Shan Z., Xie J., Yang Y. (2024). The Role of Short Chain Fatty Acids in Inflammation and Body Health. Int. J. Mol. Sci..

[37] Dharmarathne G., Kazi S., King S., Jayasinghe T.N. (2024). The Bidirectional Relationship between Cardiovascular Medications and Oral and Gut Microbiome Health: A Comprehensive Review. Microorganisms.

[38] Abdulrahim A.O., Doddapaneni N.S.P., Salman N., Giridharan A., Thomas J., Sharma K., Abboud E., Rochill K., Shreelakshmi B., Gupta V. (2025). The Gut–Heart Axis: A Review of Gut Microbiota, Dysbiosis, and Cardiovascular Disease Development. Ann. Med. Surg..

[39] Zhu G., Man J. (2026). Gut Microbiota-Derived Metabolites in Cardiovascular Disease: Mechanisms, Disease-Specific Roles, and Translational Opportunities. Front. Cardiovasc. Med..

[40] Zhu W., Gregory J.C., Org E., Buffa J.A., Gupta N., Wang Z., Li L., Fu X., Wu Y., Mehrabian M. (2016). Gut Microbial Metabolite TMAO Enhances Platelet Hyperreactivity and Thrombosis Risk. Cell.

[41] Li D., Lu Y., Yuan S., Cai X., He Y., Chen J., Wu Q., He D., Fang A., Bo Y. (2022). Gut microbiota–derived metabolite trimethylamine-N-oxide and multiple health outcomes: An umbrella review and updated meta-analysis. Am. J. Clin. Nutr..

[42] Li F., Tai L., Sun X., Lv Z., Tang W., Wang T., Zhao Z., Gong D., Ma S., Tang S. (2024). Molecular recognition and activation mechanism of short-chain fatty acid receptors FFAR2/3. Cell Res..

[43] Hodgkinson K., El Abbar F., Dobranowski P., Manoogian J., Butcher J., Figeys D., Mack D., Stintzi A. (2023). Butyrate’s role in human health and the current progress towards its clinical application to treat gastrointestinal disease. Clin. Nutr..

[44] Lu Y., Zhang Y., Zhao X., Shang C., Xiang M., Li L., Cui X. (2022). Microbiota-derived short-chain fatty acids: Implications for cardiovascular and metabolic disease. Front. Cardiovasc. Med..

[45] Neuschwander-Tetri B.A., Loomba R., Sanyal A.J., Lavine J.E., Van Natta M.L., Abdelmalek M.F., Chalasani N., Dasarathy S., Diehl A.M., Hameed B. (2015). Farnesoid X nuclear receptor ligand obeticholic acid for non-cirrhotic, non-alcoholic steatohepatitis (FLINT): A multicentre, randomised, placebo-controlled trial. Lancet.

[46] Lu Q., Chen J., Jiang L., Geng T., Tian S., Liao Y., Yang K., Zheng Y., He M., Tang H. (2024). Gut microbiota-derived secondary bile acids, bile acids receptor polymorphisms, and risk of cardiovascular disease in individuals with newly diagnosed type 2 diabetes: A cohort study. Am. J. Clin. Nutr..

[47] Gao P., Rinott E., Dong D., Mei Z., Wang F., Liu Y., Kamer O., Yaskolka Meir A., Tuohy K.M., Blüher M. (2024). Gut microbial metabolism of bile acids modifies the effect of Mediterranean diet interventions on cardiometabolic risk in a randomized controlled trial. Gut Microbes.

[48] Violi F., Cammisotto V., Bartimoccia S., Pignatelli P., Carnevale R., Nocella C. (2023). Gut-derived low-grade endotoxaemia, atherothrombosis and cardiovascular disease. Nat. Rev. Cardiol..

[49] Munford R.S. (2016). Endotoxemia-menace, marker, or mistake?. J. Leukoc. Biol..

[50] Xue H., Chen X., Yu C., Deng Y., Zhang Y., Chen S., Chen X., Chen K., Yang Y., Ling W. (2022). Gut Microbially Produced Indole-3-Propionic Acid Inhibits Atherosclerosis by Promoting Reverse Cholesterol Transport and Its Deficiency Is Causally Related to Atherosclerotic Cardiovascular Disease. Circ. Res..

[51] Nemet I., Saha P.P., Gupta N., Zhu W., Romano K.A., Skye S.M., Cajka T., Mohan M.L., Li L., Wu Y. (2020). A Cardiovascular Disease-Linked Gut Microbial Metabolite Acts via Adrenergic Receptors. Cell.

[52] Gui D.D., Luo W., Yan B.J., Ren Z., Tang Z.H., Liu L.S., Zhang J.F., Jiang Z.S. (2021). Effects of gut microbiota on atherosclerosis through hydrogen sulfide. Eur. J. Pharmacol..

[53] Tobias D.K., Lawler P.R., Harada P.H., Demler O.V., Ridker P.M., Manson J.E., Cheng S., Mora S. (2018). Circulating Branched-Chain Amino Acids and Incident Cardiovascular Disease in a Prospective Cohort of US Women. Circ. Genom. Precis. Med..

[54] Jiang W., Lu K., Zhuang Z., Wang X., Tang X., Huang T., Gao P., Wang Y., Du J. (2023). Mendelian Randomization Analysis Provides Insights into the Pathogenesis of Serum Levels of Branched-Chain Amino Acids in Cardiovascular Disease. Metabolites.

[55] Karlsson F.H., Fåk F., Nookaew I., Tremaroli V., Fagerberg B., Petranovic D., Bäckhed F., Nielsen J. (2012). Symptomatic atherosclerosis is associated with an altered gut metagenome. Nat. Commun..

[56] He J.H., Wang H., Qiu E., Qi Q., Wang Z. (2025). Gut Microbiota and Atherosclerosis: Integrative Multi-Omics and Mechanistic Insights. Curr. Atheroscler. Rep..

[57] Yan Q., Gu Y., Li X., Yang W., Jia L., Chen C., Han X., Huang Y., Zhao L., Li P. (2017). Alterations of the Gut Microbiome in Hypertension. Front. Cell. Infect. Microbiol..

[58] Cai M., Lin L., Jiang F., Peng Y., Li S., Chen L., Lin Y. (2023). Gut microbiota changes in patients with hypertension: A systematic review and meta-analysis. J. Clin. Hypertens..

[59] Emoto T., Hayashi T., Tabata T., Yamashita T., Watanabe H., Takahashi T., Gotoh Y., Kami K., Yoshida N., Saito Y. (2021). Metagenomic analysis of gut microbiota reveals its role in trimethylamine metabolism in heart failure. Int. J. Cardiol..

[60] Tang W.H.W., Wang Z., Fan Y., Levison B., Hazen J.E., Donahue L.M., Wu Y., Hazen S.L. (2014). Prognostic value of elevated levels of intestinal microbe-generated metabolite trimethylamine-N-oxide in patients with heart failure: Refining the gut hypothesis. J. Am. Coll. Cardiol..

[61] Trøseid M., Ueland T., Hov J.R., Svardal A., Gregersen I., Dahl C.P., Aakhus S., Gude E., Bjørndal B., Halvorsen B. (2015). Microbiota-dependent metabolite trimethylamine-N-oxide is associated with disease severity and survival of patients with chronic heart failure. J. Intern. Med..

[62] Li W., Huang A., Zhu H., Liu X., Huang X., Huang Y., Cai X., Lu J., Huang Y. (2020). Gut microbiota-derived trimethylamine N-oxide is associated with poor prognosis in patients with heart failure. Med. J. Aust..

[63] Zuo K., Yin X., Li K., Zhang J., Wang P., Jiao J., Liu Z., Liu X., Liu J., Li J. (2020). Different Types of Atrial Fibrillation Share Patterns of Gut Microbiota Dysbiosis. mSphere.

[64] Zuo K., Liu X., Wang P., Jiao J., Han C., Liu Z., Yin X., Li J., Yang X. (2020). Metagenomic data-mining reveals enrichment of trimethylamine-N-oxide synthesis in gut microbiome in atrial fibrillation patients. BMC Genom..

[65] Mylavarapu M., Tiwari A., Kaur H., Vempati R., Kumar H., Kodali L.S.M., Khan K.G., Dadana S., Garcia I., Cabrera F.E.P. (2026). The Gut-Heart Axis: A Comprehensive Review of Microbiota’s Role in Cardiovascular Health and Disease and Emerging Therapeutic Strategies. Cardiol. Res. Pract..

[66] Ahmad I., Sivakumar M., Singla S., Singla B., Kumawat S., Ali G., Raza Mehdi A. (2026). Effects of Gut Microbiota-Modulating Interventions on Systemic Inflammation and Functional Outcomes in Established Cardiovascular Disease: A Systematic Review. Cureus.

[67] Wang D.D., Nguyen L.H., Li Y., Yan Y., Ma W., Rinott E., Ivey K.L., Shai I., Willett W.C., Hu F.B. (2021). The gut microbiome modulates the protective association between a Mediterranean diet and cardiometabolic disease risk. Nat. Med..

[68] García-Gavilán J.F., Atzeni A., Babio N., Liang L., Belzer C., Vioque J., Corella D., Fitó M., Vidal J., Moreno-Indias I. (2024). Effect of 1-year lifestyle intervention with energy-reduced Mediterranean diet and physical activity promotion on the gut metabolome and microbiota: A randomized clinical trial. Am. J. Clin. Nutr..

[69] Yu J., Wu Y., Zhu Z., Lu H. (2025). The impact of dietary patterns on gut microbiota for the primary and secondary prevention of cardiovascular disease: A systematic review. Nutr. J..

[70] Shremo Msdi A., Wang E.M., Garey K.W. (2025). Prebiotics Improve Blood Pressure Control by Modulating Gut Microbiome Composition and Function: A Systematic Review and Meta-Analysis. Nutrients.

[71] Talukdar J.R., Cooper M., Lyutvyn L., Zeraatkar D., Ali R., Berbrier R., Janes S., Ha V., Darling P.B., Xue M. (2024). The effects of inulin-type fructans on cardiovascular disease risk factors: Systematic review and meta-analysis of randomized controlled trials. Am. J. Clin. Nutr..

[72] da C. Pinaffi-Langley A.C., Tarantini S., Hord N.G., Yabluchanskiy A. (2024). Polyphenol-Derived Microbiota Metabolites and Cardiovascular Health: A Concise Review of Human Studies. Antioxidants.

[73] Cano R., Bermúdez V., Galban N., Garrido B., Santeliz R., Gotera M.P., Duran P., Boscan A., Carbonell-Zabaleta A.K., Durán-Agüero S. (2024). Dietary Polyphenols and Gut Microbiota Cross-Talk: Molecular and Therapeutic Perspectives for Cardiometabolic Disease: A Narrative Review. Int. J. Mol. Sci..

[74] Mishra A.K., Singh R., Rawat H., Kumar V., Jagtap C., Jain A. (2024). The influence of food matrix on the stability and bioavailability of phytochemicals: A comprehensive review. Food Humanit..

[75] O’Sullivan E., Solano O., Oliveira J.S., Johnson A.J., Dhital S., Pillai P.K.S., Dias F., Ubbink J., Lin A.W., Teigen L. (2026). The food matrix as a confounder in diet–microbiome studies: Methodological challenges and research gaps. Gut Microbes Rep..

[76] Sejbuk M., Mirończuk-Chodakowska I., Karav S., Witkowska A.M. (2024). Dietary Polyphenols, Food Processing and Gut Microbiome: Recent Findings on Bioavailability, Bioactivity, and Gut Microbiome Interplay. Antioxidants.

[77] Hernández-Bautista M., Gutiérrez T.J., Tovar J., Bello-Pérez L.A. (2025). Effect of starch structuring and processing on the bioaccessibility of polyphenols in starchy foodstuffs: A review. Food Res. Int..

[78] Hill C., Guarner F., Reid G., Gibson G.R., Merenstein D.J., Pot B., Morelli L., Canani R.B., Flint H.J., Salminen S. (2014). Expert consensus document. The International Scientific Association for Probiotics and Prebiotics consensus statement on the scope and appropriate use of the term probiotic. Nat. Rev. Gastroenterol. Hepatol..

[79] Aditya M.R., Hogipranata M.O., Simanjuntak A.M.T., Rampengan D.D.C.H., Kizzandy K., Karolina W., Pramudya A., Sulistomo H.W. (2026). Modulating Gut Microbiota To Control Hypertension: A Systematic Review and Meta-Analysis of Probiotics, Postbiotics, Prebiotics and Synbiotics. Probiotics Antimicrob. Proteins.

[80] Maia L.A., de Souza J.R., da Silva L.d.F.R., Magnani M., de Souza E.L., de Brito Alves J.L. (2025). Effects of Probiotics on Inflammatory Biomarkers and Its Associations With Cardiac Autonomic Function in Women With Arterial Hypertension: A Secondary Analysis of a Randomized Clinical Trial. Probiotics Antimicrob. Proteins.

[81] Dixon A., Robertson K., Yung A., Que M., Randall H., Wellalagodage D., Cox T., Robertson D., Chi C., Sun J. (2020). Efficacy of Probiotics in Patients of Cardiovascular Disease Risk: A Systematic Review and Meta-analysis. Curr. Hypertens. Rep..

[82] Dong J., Chen L., Zheng N., Yang M. (2025). The beneficial effects of probiotics on patients with coronary heart disease: A systematic review and meta-analysis. Front. Nutr..

[83] Yang X., Yu Y., Huang X., Huang J., Xiang Q., Yu R. (2026). Effects of probiotics on lipid metabolism, oxidation, and inflammation in coronary heart disease: A systematic review and meta-analysis. Front. Nutr..

[84] Mohsenzadeh A., Pourasgar S., Mohammadi A., Nazari M., Nematollahi S., Karimi Y., Firoozbakhsh P., Mohsenzadeh H., Kamali K., Elahi R. (2025). The gut microbiota and cardiovascular disease: Exploring the role of microbial dysbiosis and metabolites in pathogenesis and therapeutics. Life Sci..

[85] Gibson G.R., Hutkins R., Sanders M.E., Prescott S.L., Reimer R.A., Salminen S.J., Scott K., Stanton C., Swanson K.S., Cani P.D. (2017). Expert consensus document: The International Scientific Association for Probiotics and Prebiotics (ISAPP) consensus statement on the definition and scope of prebiotics. Nat. Rev. Gastroenterol. Hepatol..

[86] Holmes Z.C., Villa M.M., Durand H.K., Jiang S., Dallow E.P., Petrone B.L., Silverman J.D., Lin P.-H., David L.A. (2022). Microbiota responses to different prebiotics are conserved within individuals and associated with habitual fiber intake. Microbiome.

[87] Kok C.R., Rose D., Hutkins R. (2023). Predicting Personalized Responses to Dietary Fiber Interventions: Opportunities for Modulation of the Gut Microbiome to Improve Health. Annu. Rev. Food Sci. Technol..

[88] Swanson K.S., Gibson G.R., Hutkins R., Reimer R.A., Reid G., Verbeke K., Scott K.P., Holscher H.D., Azad M.B., Delzenne N.M. (2020). The International Scientific Association for Probiotics and Prebiotics (ISAPP) consensus statement on the definition and scope of synbiotics. Nat. Rev. Gastroenterol. Hepatol..

[89] Skonieczna-Żydecka K., Kaźmierczak-Siedlecka K., Kaczmarczyk M., Śliwa-Dominiak J., Maciejewska D., Janda K., Stachowska E., Łoniewska B., Malinowski D., Borecki K. (2020). The Effect of Probiotics and Synbiotics on Risk Factors Associated with Cardiometabolic Diseases in Healthy People-A Systematic Review and Meta-Analysis with Meta-Regression of Randomized Controlled Trials. J. Clin. Med..

[90] Salminen S., Collado M.C., Endo A., Hill C., Lebeer S., Quigley E.M.M., Sanders M.E., Shamir R., Swann J.R., Szajewska H. (2021). The International Scientific Association of Probiotics and Prebiotics (ISAPP) consensus statement on the definition and scope of postbiotics. Nat. Rev. Gastroenterol. Hepatol..

[91] Pimentel T.C., Cruz A.G., Pereira E., da Costa W.K.A., Rocha R.d.S., Pedrosa G.T.d.S., Rocha C.d.S., Alves J.M., Alvarenga V.O., Sant’Ana A.S. (2023). Postbiotics: An overview of concepts, inactivation technologies, health effects, and driver trends. Trends Food Sci. Technol..

[92] Trymers M., Wiśniewski P., Tkacz K., Zakrzewski A. (2026). From Production to Application: Postbiotics in Meat, Meat Products, Other Food Matrices, and Bioactive Packaging. Foods.

[93] Flori L., Benedetti G., Martelli A., Calderone V. (2024). Microbiota alterations associated with vascular diseases: Postbiotics as a next-generation magic bullet for gut-vascular axis. Pharmacol. Res..

[94] Pakmehr A., Mousavi S.M., Ejtahed H.S., Hoseini-Tavassol Z., Siadat S.D., Hasani-Ranjbar S., Larijani B. (2024). The Effect of Fecal Microbiota Transplantation on Cardiometabolic Risk Factors: A Systematic Review and Meta-Analysis. Clin. Ther..

[95] da Ponte Neto A.M., Clemente A.C.O., Rosa P.W., Ribeiro I.B., Funari M.P., Nunes G.C., Moreira L., Sparvoli L.G., Cortez R., Taddei C.R. (2023). Fecal microbiota transplantation in patients with metabolic syndrome and obesity: A randomized controlled trial. World J. Clin. Cases.

[96] Porcari S., Benech N., Valles-Colomer M., Segata N., Gasbarrini A., Cammarota G., Sokol H., Ianiro G. (2023). Key determinants of success in fecal microbiota transplantation: From microbiome to clinic. Cell Host Microbe.

[97] Dannenberg L., Zikeli D., Benkhoff M., Ahlbrecht S., Kelm M., Levkau B., Polzin A. (2020). Targeting the human microbiome and its metabolite TMAO in cardiovascular prevention and therapy. Pharmacol. Ther..

[98] Oktaviono Y.H., Dyah Lamara A., Saputra P.B.T., Arnindita J.N., Pasahari D., Saputra M.E., Suasti N.M.A. (2023). The roles of trimethylamine-N-oxide in atherosclerosis and its potential therapeutic aspect: A literature review. Biomol. Biomed..

[99] Wang Z., Roberts A.B., Buffa J.A., Levison B.S., Zhu W., Org E., Gu X., Huang Y., Zamanian-Daryoush M., Culley M.K. (2015). Non-lethal Inhibition of Gut Microbial Trimethylamine Production for the Treatment of Atherosclerosis. Cell.

[100] Zhang N., Wang Y., Luo G., Gao X. (2026). Gut Microbial Choline TMA-Lyase CutC: From Metabolic Mechanism to a Novel Therapeutic Target for Diseases. Nutrients.

[101] Zimmermann M., Zimmermann-Kogadeeva M., Wegmann R., Goodman A.L. (2019). Mapping human microbiome drug metabolism by gut bacteria and their genes. Nature.

[102] Zimmermann M., Zimmermann-Kogadeeva M., Wegmann R., Goodman A.L. (2019). Separating host and microbiome contributions to drug pharmacokinetics and toxicity. Science.

[103] Zimmermann M., Patil K.R., Typas A., Maier L. (2021). Towards a mechanistic understanding of reciprocal drug-microbiome interactions. Mol. Syst. Biol..

[104] Zimmermann F., Roessler J., Schmidt D., Jasina A., Schumann P., Gast M., Poller W., Leistner D., Giral H., Kränkel N. (2020). Impact of the Gut Microbiota on Atorvastatin Mediated Effects on Blood Lipids. J. Clin. Med..

[105] Wilmanski T., Kornilov S.A., Diener C., Conomos M.P., Lovejoy J.C., Sebastiani P., Orwoll E.S., Hood L., Price N.D., Rappaport N. (2022). Heterogeneity in statin responses explained by variation in the human gut microbiome. Med.

[106] Dias A.M., Cordeiro G., Estevinho M.M., Veiga R., Figueira L., Reina-Couto M., Magro F., the Clinical Pharmacology Unit, São João Hospital University Centre (2020). Gut bacterial microbiome composition and statin intake-A systematic review. Pharmacol. Res. Perspect..

[107] Wang E.M., Shremo Msdi A., Quach V.N., Nguyen S.Q., Quach E., Jo J., Eubank T.A., Garey K.W., Rosario N. (2025). Angiotensin converting enzyme inhibitors and angiotensin receptor blockers impact on the gut microbiome: A systematic review. Front. Endocrinol..

[108] Li J., Wang S.-Y., Yan K.-X., Wang P., Jiao J., Wang Y.-D., Chen M.-L., Dong Y., Zhong J.-C. (2024). Intestinal Microbiota by Angiotensin Receptor Blocker Therapy Exerts Protective Effects Against Hypertensive Damages. iMeta.

[109] Deng X., Zhang C., Wang P., Wei W., Shi X., Wang P., Yang J., Wang L., Tang S., Fang Y. (2022). Cardiovascular Benefits of Empagliflozin Are Associated With Gut Microbiota and Plasma Metabolites in Type 2 Diabetes. J. Clin. Endocrinol. Metab..

[110] Li T., Ding N., Guo H., Hua R., Lin Z., Tian H., Yu Y., Fan D., Yuan Z., Gonzalez F.J. (2024). A gut microbiota-bile acid axis promotes intestinal homeostasis upon aspirin-mediated damage. Cell Host Microbe.

[111] Chen G., Wang Z., Song W., Liao Y., Wang X., Chen C., Ming J., Cui J., Xu K. (2023). Effects of long-term regular oral aspirin combined with atorvastatin to prevent ischemic stroke on human gut microbiota. Eur. J. Pharmacol..

[112] Skye S.M., Zhu W., Romano K.A., Guo C.J., Wang Z., Jia X., Kirsop J., Haag B., Lang J.M., DiDonato J.A. (2018). Microbial Transplantation With Human Gut Commensals Containing CutC Is Sufficient to Transmit Enhanced Platelet Reactivity and Thrombosis Potential. Circ. Res..

[113] Amin A.M., Sheau Chin L., Teh C.H., Mostafa H., Mohamed Noor D.A., Abdul Kader M.A.S.K., Kah Hay Y., Ibrahim B. (2018). Pharmacometabolomics analysis of plasma to phenotype clopidogrel high on treatment platelets reactivity in coronary artery disease patients. Eur. J. Pharm. Sci..

[114] Gilbert J.A., Azad M.B., Bäckhed F., Blaser M.J., Byndloss M., Chiu C.Y., Chu H., Dugas L.R., Elinav E., Gibbons S.M. (2025). Clinical translation of microbiome research. Nat. Med..

[115] Schierwagen R., Carraturo F., Iyappan A., Brol M.J., Druart C., Israelsen M., Hassani Z., Legent K., Godoy Y., Vecchi C. (2026). Multidisciplinary Delphi consensus statement on minimal standards for clinical metadata and end points in microbiome studies. Nat. Rev. Gastroenterol. Hepatol..

[116] Shi B., Li H., He X. (2024). Advancing lifelong precision medicine for cardiovascular diseases through gut microbiota modulation. Gut Microbes.

[117] Kumar A., Xu C., Dakal T.C. (2026). Microbiome based precision medicine through integrated multiomics and machine learning. Microbiol. Res..

[118] Mamic P., Snyder M., Tang W.H.W. (2023). Gut Microbiome-Based Management of Patients With Heart Failure: JACC Review Topic of the Week. J. Am. Coll. Cardiol..

[119] Rozera T., Pasolli E., Segata N., Ianiro G. (2025). Machine Learning and Artificial Intelligence in the Multi-Omics Approach to Gut Microbiota. Gastroenterology.

[120] Montazeri-Najafabady N. (2026). From One-Size-Fits-All to Precision Medicine: The Promise of Personalized Probiotics. Probiotics Antimicrob. Proteins.

[121] Jiang Y., Jiang S., Wang Z., Zhu P., Zhang J., Teng F., Huang S. (2026). Precise probiotic therapy: Advances, bottlenecks, and the road to microbiome-informed nutrition. Gut Microbes.

[122] Khera R., Oikonomou E.K., Nadkarni G.N., Morley J.R., Wiens J., Butte A.J., Topol E.J. (2024). Transforming Cardiovascular Care With Artificial Intelligence: From Discovery to Practice: JACC State-of-the-Art Review. J. Am. Coll. Cardiol..

[123] Metwaly A., Kriaa A., Hassani Z., Carraturo F., Druart C., Arnauts K., Wilmes P., Walter J., Rosshart S., Desai M.S. (2025). A Consensus Statement on establishing causality, therapeutic applications and the use of preclinical models in microbiome research. Nat. Rev. Gastroenterol. Hepatol..

